# Effect of sugammadex on postoperative complications in patients with severe burn who underwent surgery: a retrospective study

**DOI:** 10.1038/s41598-024-51171-y

**Published:** 2024-01-04

**Authors:** Jong Ho Kim, Minguan Kim, Minho Oh, Soo-Kyung Lee, Young Suk Kwon

**Affiliations:** 1 Department of Anesthesiology and Pain Medicine, College of Medicine, Chuncheon Sacred Heart Hospital, Hallym University, 77 Sakju-ro, Chuncheon, 24253 South Korea; 2 Institute of New Frontier Research Team, Hallym University, Chuncheon, South Korea; 3 Department of Anesthesiology and Pain Medicine, College of Medicine, Hallym University Sacred Heart Hospital, Hallym University, Anyang, Republic of Korea

**Keywords:** Diseases, Risk factors

## Abstract

This retrospective study investigated the association of sugammadex with postoperative pulmonary complication risk between 2013 and 2021 in patients with severe burn of five hospitals. Postoperative pulmonary complications included atelectasis, pulmonary edema, pulmonary effusion, pneumothorax, pneumonia, pulmonary thromboembolism, respiratory failure and acute respiratory distress. To identify whether sugammadex reduced the risk of postoperative pulmonary complication in patients with severe burn who underwent surgery, Kaplan–Meier curve were used to check the difference of incidence according to surgical cases and time-varying Cox hazard regression were used to calculate the hazard ratio. The study included 1213 patients with severe burn who underwent 2259 surgeries. Postoperative pulmonary complications were occurred in 313 (25.8%) patients. Among 2259 surgeries, sugammadex was used in 649 (28.7%) surgeries. Cumulative postoperative pulmonary complication were 268 (16.6%) cases in surgeries without sugammadex, and 45 (6.9%) cases in surgeries with sugammadex, respectively (P < 0.005). The postoperative pulmonary complications risk was reduced significantly in patients who use sugammadex than those who did not use sugammadex. (Adjusted hazard ratio, 0.61; 95% confidence interval, 0.42–0.89; P = 0.011). In conclusion, sugammadex reduced risk of postoperative pulmonary complications compared with nonuse of sugammadex in patients with severe burn who underwent surgery.

## Introduction

Burns affect nearly 500,000 people worldwide each year, and burns caused by severe trauma have a prevalence of 0.4% to 5.8%. These injuries pose a multifaceted challenge due to their significant physiological effects and the potential for serious complications resulting from the severe breakdown of skin integrity and multiple organ functions^1,2^. The pathophysiological changes induced by burn injuries are significant, disrupting essential mechanisms such as thermal regulation, fluid and electrolyte balance, and immune defenses against bacterial infections^3^. Notably, burns trigger alterations in vascular permeability, causing systemic effects like increased edema and compromised vascular integrity in regions remote from the initial burn site^4^. Furthermore, the ramifications of burns extend beyond local tissue damage, impacting respiratory function substantially. These injuries lead to reductions in functional residual capacity, lung capacity, and chest wall compliance, accompanied by an elevated alveolar–arterial oxygen gradient and minute ventilation. These changes significantly heighten the risk of developing pulmonary complications, including hypoventilation and respiratory failure^5^.

In addressing burn injuries, early aggressive surgical interventions such as debridement and closure of nonviable tissue play a pivotal role. These interventions are crucial for reducing infection rates and expediting the healing process^6,7^. Consequently, many burn patients often require multiple surgeries aimed at both functional restoration and aesthetic enhancement, essential components in managing acute burn injuries and mitigating subsequent complications^8^. However, this intensive surgical intervention often raises concerns about the heightened risk of postoperative pulmonary complications, reported in 5–9% of burn patients, depending on the criteria used for defining complications^9^.

Moreover, burn injuries have a notable impact on neuromuscular function, potentially influencing the development of postoperative pulmonary complications. Abnormal responses to neuromuscular blocking agents in burn patients, attributed to alterations in nicotinic acetylcholine receptor regulation and changes in plasma protein binding, pose challenges in the usage and reversal of these agents^10^. In this context, sugammadex, a modified γ-cyclodextrin, emerges as a potential solution. It acts as a reversal agent for steroidal non-depolarizing neuromuscular blocking agents, ensuring swift and predictable recovery from neuromuscular blockade^11^. Previous large cohort studies and bariatric surgery studies have suggested the potential benefits of sugammadex in reducing postoperative pulmonary complications^12,13^. However, despite these indications, no investigations have examined the specific effects of sugammadex in severe burn patients. By investigating this underexplored area, our study hypothesizes that sugammadex administration may mitigate postoperative pulmonary complications in severe burn patients, contributing valuable insights into improving surgical outcomes in this unique patient population. This study aims to fill this critical gap by exploring the impact of sugammadex on the risk of postoperative complications in severe burn patients undergoing surgery.

## Results

### Cohort characteristics

**Figure 1:**
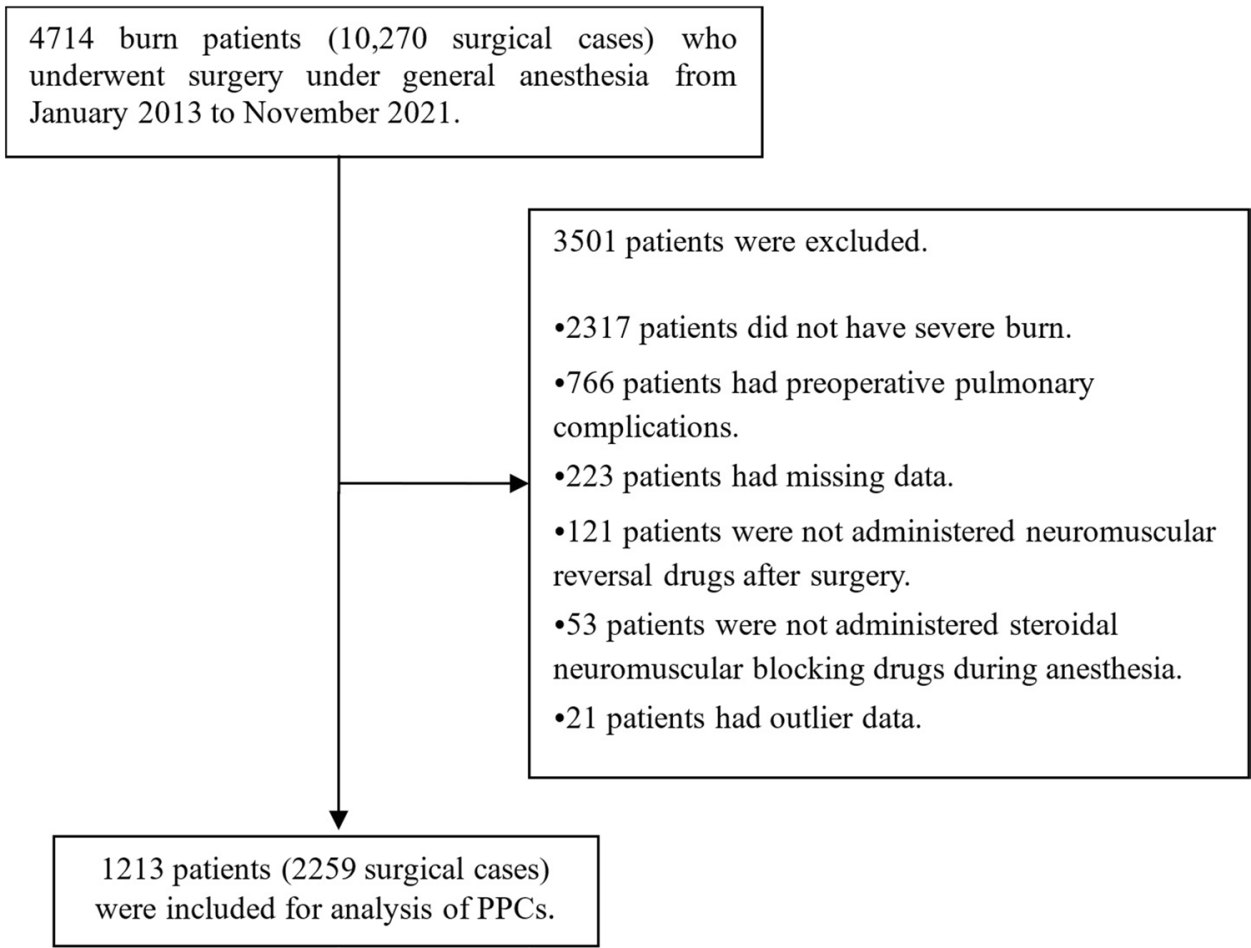
Flowchart of patient selection.

During the investigation conducted from January 1, 2013, to November 15, 2021, a total of 4714 burn patients underwent 10,270 surgeries at research institutions. Following the application of exclusion criteria, 1213 severe burn patients were included, accounting for 2259 surgeries in the study. These patients were analyzed to assess potential disparities in the occurrence of postoperative pulmonary complications based on the administration or non-administration of sugammadex to severe burn patients undergoing surgery (as illustrated in Fig. 1). Among all patients, postoperative pulmonary complications were observed in 313 cases, accounting for 25.3% of the total patient population and occurring in 13.9% of all surgeries. Sugammadex was utilized in 649 cases (28.7%) out of the total 2259 surgeries performed on severe burn patients. Table 1 summarizes the burn characteristics of patients with severe burns who underwent surgery under general anesthesia, categorized by the presence or absence of postoperative pulmonary complications. Chi-square test or Fisher’s exact test was employed to examine the differences in burn characteristics between those with and without postoperative pulmonary complications.

**Table 1:** Burn characteristics in patients with severe burn who underwent surgery.

Characteristics of burn	No PPC (n = 900)	PPC (n = 313)	P value
Head and neck (2nd grade/3rd grade)	231 (25.7)/44 (4.9)	117 (37.4)/37 (11.8)	< 0.001^†^
Trunk (2nd grade/3rd grade)	167 (18.6)/123 (13.7)	83 (26.5)/85 (27.2)	< 0.001^†^
Shoulder and upper limb, except wrist and hand (2nd grade/3rd grade)	165 (18.3)/164 (18.2)	47 (15.0)/77 (24.6)	0.003^†^
Wrist and hand (2nd grade/3rd grade)	202 (22.4)/265 (29.4)	65 (20.8)/91 (29.1)	0.7353^†^
Hip and lower limb, except ankle and foot (2nd grade/3rd grade)	174 (19.3)/149 (16.6)	40 (12.8)/105 (33.5)	< 0.001^†^
Ankle and foot (2nd grade/3rd grade)	73 (8.1)/108 (12.0)	19 (6.1)/52 (16.6)	0.013^†^
Eye and adnexa	13 (1.4)	3 (1.0)	0.772*
Respiratory tract	28 (3.1)	24 (7.7)	< 0.001^†^
Other internal organs	1 (0.1)	1 (0.3)	0.5*
Chemical burn	142 (15.8)	5 (1.6)	< 0.001*
Electrical burn	387 (43.0)	53 (16.9)	< 0.001^†^
Burn size grade	1.0 (0.0, 2.0)	3.0 (2.0, 5.0)	< 0.001^†^

Data are presented as numbers (%) or medians (interquartile range).

Burn size grade: 0, < 10% of TBS; 1, 10–19% of TBS; 2, 20–29% of TBS; 3, 30–39% of TBS; 5, 50–59% of TBS.

*PPC* postoperative pulmonary complication, *TBS* total body surface.

^†^Chi-square test.

*Fisher’s exact test.

Tables 2 and 3 present the demographic characteristics and perioperative data of the patients, respectively, distinguished by the use or non-use of sugammadex. Chi-square test, Fisher’s exact test, or Mann–Whitney U test was utilized to explore the dissimilarities in characteristics between patients who received sugammadex and those who did not.

**Table 2:** Demographic characteristics in surgical cases of patients with severe burn who underwent surgery.

	Nonuse of sugammadex (n = 1610)	Sugammadex (n = 649)	P value
Old age (≥ 70 years)	139 (8.6)	65 (10.0)	0.339^†^
Male	1382 (85.8)	555 (85.5)	0.895^†^
Obesity (BMI ≥ 30)	53 (3.3)	30 (4.6)	0.162^†^
Congestive heart failure	16 (1.0)	1 (0.1)	0.055*
Cardiac arrhythmias	16 (1.0)	12 (1.9)	0.146^†^
Valvular disease	7 (0.4)	2 (0.3)	> 0.999
Pulmonary circulation disorders	2 (0.1)	2 (0.3)	0.326*
Peripheral vascular disorders	8 (0.5)	0 (0)	0.115*
Hypertension, uncomplicated	140 (8.7)	50 (7.7)	0.494^†^
Hypertension, complicated	46 (2.9)	7 (1.1)	0.013*
Paralysis	8 (0.5)	6 (0.9)	0.245*
Other neurological disorders	8 (0.5)	4 (0.6)	0.752*
Chronic pulmonary disease	12 (0.8)	1 (0.1)	0.126*
Diabetes, uncomplicated	30 (1.9)	18 (2.8)	0.232^†^
Diabetes, complicated	334 (20.8)	55 (8.5)	< 0.001^†^
Hypothyroidism	3 (0.2)	0 (0)	0.562*
Renal failure	10 (0.6)	3 (0.5)	0.768*
Liver disease	45 (2.8)	22 (3.4)	0.537^†^
Peptic ulcer disease excluding bleeding	8 (0.5)	1 (0.1)	0.461*
Coagulopathy	4 (0.2)	1 (0.1)	> 0.999*
Fluid and electrolyte disorders	1 (0.1)	0 (0)	> 0.999*
Deficiency anemia	3 (0.2)	1 (0.1)	> 0.999*
Alcohol abuse	1 (0.1)	1 (0.1)	0.492*
Drug abuse	9 (0.6)	4 (0.6)	> 0.999*
Psychoses	9 (0.6)	6 (0.9)	0.39*
Depression	218 (13.5)	101 (15.6)	0.237^†^
Alcohol	867 (53.9)	364 (56.1)	0.358^†^
Smoking	645 (40.1)	254 (39.1)	0.72^†^

*BMI* body mass index.

^†^Chi-square test.

*Fisher’s exact test.

**Table 3:** Perioperative data in surgical cases of patients with severe burn who underwent surgery.

	Nonuse of sugammadex (n = 1610)	Sugammadex (n = 649)	P value
Emergency	96 (6.0)	48 (7.4)	0.243^‡^
ASA PS > 2	271 (16.8)	102 (15.7)	0.559^‡^
N_2_O	1514 (94.0)	626 (96.5)	0.026^‡^
Inhalation anesthetics	1536 (95.4)	643 (99.1)	< 0.001*
Rocuronium (mg)	100.0 (50.0, 100.0)	100.0 (100.0, 100.0)	< 0.001^‖^
Surgery time (h)	1.3 (0.8, 2.1)	1.2 (0.8, 1.8)	0.195^‖^
Intraoperative fluid (L)	0.8 (0.4, 1.6)	0.6 (0.2, 1.1)	< 0.001^‖^
Estimated blood loss (L)	0.3 (0.1, 0.6)	0.1 (0.0, 0.3)	< 0.001^‖^
Arterial line monitoring	708 (44.0)	280 (43.1)	0.754^‡^
Central venous line monitoring	17 (1.1)	1 (0.1)	0.033*
Foley catheter	784 (48.7)	289 (44.5)	0.081^‡^
Patient controlled analgesia	1280 (79.5)	561 (86.4)	< 0.001^‡^
Intraoperative PRBC (unit)	0.0 (0.0, 3.0)	0.0 (0.0, 2.0)	0.02^‖^
BUN > 23 mg/dL	63 (3.9)	20 (3.1)	0.408^‡^
Creatinine > 1.2 mg/dL	55 (3.4)	17 (2.6)	0.399^‡^
aPTT > 40 s	358 (22.2)	201 (31.0)	< 0.001^‡^
PT, INR > 1.2	171 (10.6)	30 (4.6)	< 0.001^‡^
Platelet < 50,000 μL^−1^	12 (0.8)	0 (0)	0.024*
Albumin < 3.5 g/dL	83 (5.2)	9 (1.4)	< 0.001*
Abdomen surgery	7 (0.4)	1 (0.1)	0.452*
Musculoskeletal surgery	273 (17.0)	127 (19.6)	0.158^‡^
Neurosurgery	2 (0.1)	3 (0.5)	0.146*
OBGY surgery	1 (0.1)	0 (0)	> 0.999*
Spine surgery	1 (0.1)	0 (0)	> 0.999*
Thoracic surgery	6 (0.4)	0 (0)	0.191*
Vascular surgery	2 (0.1)	1 (0.1)	> 0.999*
Skin and soft tissue surgery	1545 (96.0)	626 (96.5)	0.668^‡^
Major operation surgery	33 (2.0)	20 (3.1)	0.189^‡^
Period from burn to surgery (day)	18.0 (6.0, 43.0)	16.0 (8.0, 35.0)	0.105^‖^
Intraoperative NSIADs	42 (2.6)	6 (0.9)	0.01*
NSAIDs in patient controlled analgesia	624 (38.8)	58 (8.9)	< 0.001^‡^
Duration of postop. AAP (day)	1 (0, 3)	0 (0, 2)	0.008^‖^
Duration of postop. Nefopam (day)	0 (0, 0)	0 (0, 0)	0.029^‖^
Duration of postop. NSAIDs (day)	1 (0, 4)	3 (0, 6.5)	< 0.001^‖^
Start date (day)	0.0 (0.0, 23.8)	6.8 (0.0, 25.1)	0.017^‖^
Stop date (day)	21.0 (11.5, 48.9)	23.7 (13.0, 45.6)	0.172^‖^

Data are presented as numbers (%) or medians (interquartile range).

*AAP* acetaminophen, *aPTT* activated partial thromboplastin time, *ASA PS* American Society of Anesthesiologists physical status, *BMI* body mass index, *BUN* blood urea nitrogen, *INR* international normalized ratio, *n* number of surgical cases, *NSAID* non-steroidal anti-inflammatory drug, *OBGY* obstetric and gynecological, *PRBC* packed red blood cell, *Preop.* preoperative, *PT* prothrombin time.

^†^Start date, start of observation based on the date of first surgery (observation start date − date of first surgery);

^††^Stop date, end of observation based on the date of first surgery (observation stop date − date of first surgery).

^‡^Chi-square test.

*Fisher’s exact test.

^‖^Mann–Whitney test.

### Cumulative rate of postoperative pulmonary complication among surgical patients according to use of sugammadex

**Figure 2:**
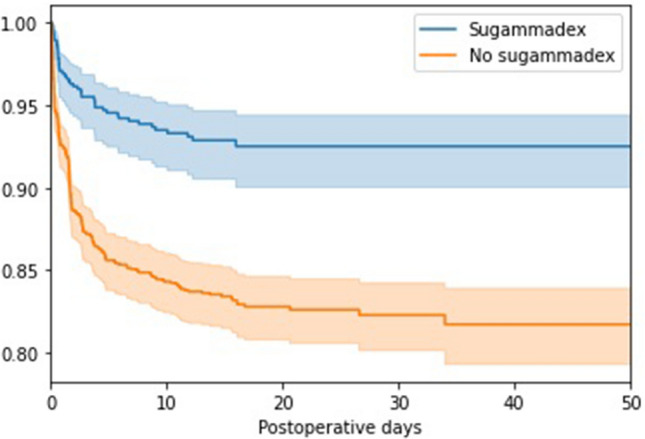
Kaplan–Meier curve for surgical cases according to use of sugammadex.

The cumulative rate of postoperative pulmonary complications among surgical patients was assessed based on the use of sugammadex. Out of 1610 surgeries where sugammadex was not administered, postoperative pulmonary complications were observed in 268 cases (16.6%) with a median onset of 1.5 days (interquartile range [IQR] of 0.3 to 2.8 days). Conversely, among the 649 surgeries where sugammadex was administered, postoperative pulmonary complications occurred in 45 instances (6.9%) with a median onset of 1.5 days (IQR of 0.6 to 4.5 days). Statistical analysis using the chi-square test revealed a highly significant association (P < 0.001) between the use of sugammadex and the occurrence of postoperative pulmonary complications. Additionally, Fig. 2 illustrates the Kaplan–Meier curve representing surgical patients based on sugammadex administration in surgery cases, showing a significant difference upon analysis using the log-rank test (P < 0.005).

### HR of sugammadex on postoperative pulmonary complications in patients with severe burn

In the analysis assessing the effect of sugammadex using time-varying Cox regression, the unadjusted HR of sugammadex for nonuse of sugammadex was 0.45 (95% CI 0.23–0.61; P < 0.001). After adjusting for other variables, the adjusted HR of sugammadex for nonuse of sugammadex was 0.61 (95% CI 0.42–0.89; P = 0.011). Table 4 provides a summary of both the unadjusted and adjusted HRs for all variables.

**Table 4:** Unadjusted and adjusted hazard ratio on developing postoperative pulmonary complications in patients with severe burn who underwent.

	Unadjusted HR (95% CI)	P value	Adjusted HR (95% CI)	P value
Old age (≥ 70 years)	2.07 (1.58–2.72)	< 0.001	1.78 (1.2–2.65)	0.005
Male	0.88 (0.67–1.16)	0.353	0.92 (0.65–1.3)	0.626
Obesity (BMI ≥ 30)	1.63 (0.99–2.7)	0.056	1.22 (0.71–2.12)	0.471
Congestive heart failure	2.75 (1.14–6.67)	0.025	1.28 (0.42–3.88)	0.668
Cardiac arrhythmias	2.48 (1.23–5)	0.011	1.9 (0.69–5.24)	0.218
Valvular disease	1.88 (0.47–7.56)	0.373	0.79 (0.1–6.14)	0.822
Pulmonary circulation disorders	2.24 (0.56–9)	0.255	17.78 (2.26–140.13)	0.006
Peripheral vascular disorders	0.81 (0.11–5.78)	0.835	8.82 (0.93–83.39)	0.058
Hypertension, uncomplicated	1.24 (0.85–1.79)	0.262	0.97 (0.56–1.7)	0.925
Hypertension, complicated	1.44 (0.77–2.7)	0.26	1.26 (0.57–2.81)	0.568
Paralysis	1.93 (0.62–6)	0.259	0.37 (0.07–2.13)	0.269
Other neurological disorders	4.23 (1.36–13.22)	0.013	3.25 (0.88–11.97)	0.076
Chronic pulmonary disease	0 (0–Inf)	0.991	0 (0–Inf)	0.992
Diabetes, uncomplicated	0.98 (0.44–2.21)	0.967	1.09 (0.36–3.29)	0.885
Diabetes, complicated	0.93 (0.69–1.27)	0.651	1.16 (0.8–1.69)	0.435
Hypothyroidism	2.75 (0.39–19.56)	0.314	3.13 (0.39–25.12)	0.282
Renal failure	2.69 (1.11–6.51)	0.028	1.04 (0.33–3.29)	0.952
Liver disease	1.03 (0.49–2.18)	0.937	1.71 (0.76–3.84)	0.197
Peptic ulcer disease excluding bleeding	0 (0–Inf)	0.988	0 (0–Inf)	0.993
Coagulopathy	6.87 (2.55–18.52)	< 0.001	0.66 (0.21–2.03)	0.467
Fluid and electrolyte disorders	3.91 (0.55–27.88)	0.173	0.35 (0.01–8.58)	0.519
Deficiency anemia	1.23 (0.17–8.77)	0.835	0.85 (0.07–9.63)	0.895
Alcohol abuse	0 (0–Inf)	0.989	0 (0–Inf)	0.997
Drug abuse	1.7 (0.55–5.31)	0.359	1.38 (0.38–4.96)	0.621
Psychoses	3.09 (1.15–8.28)	0.025	1.83 (0.63–5.34)	0.268
Depression	0.9 (0.63–1.29)	0.568	0.78 (0.51–1.18)	0.234
Alcohol	0.93 (0.75–1.16)	0.531	1.08 (0.82–1.43)	0.575
Smoking	1.03 (0.82–1.3)	0.776	0.96 (0.73–1.27)	0.782
Emergency	3 (2.27–3.97)	< 0.001	1.43 (1.01–2)	0.042
ASA PS > 2	6.8 (5.4–8.55)	< 0.001	1.54 (1.15–2.08)	0.004
N_2_O	0.28 (0.21–0.38)	< 0.001	0.7 (0.48–1.02)	0.061
Inhalation anesthetics	2.45 (1.01–5.93)	0.047	1.19 (0.44–3.17)	0.733
Rocuronium	1.01 (1.01–1.01)	< 0.001	1 (0.99–1)	0.268
Surgery time (h)	1.15 (1.06–1.24)	< 0.001	1.03 (0.93–1.15)	0.55
Fluid (L)	1.26 (1.22–1.3)	< 0.001	1.07 (0.95–1.2)	0.268
Estimated blood loss (L)	2.57 (2.27–2.9)	< 0.001	0.73 (0.53–1.02)	0.061
Arterial line monitoring	13.62 (8.97–20.67)	< 0.001	3.22 (1.85–5.59)	< 0.001
Central venous line monitoring	2.36 (1.11–4.99)	0.025	1.04 (0.46–2.38)	0.918
Foley catheter	11.79 (7.77–17.89)	< 0.001	1.88 (1.11–3.19)	0.02
Patient-controlled analgesia	0.33 (0.26–0.41)	< 0.001	0.86 (0.63–1.17)	0.33
Intraoperative PRBC (unit)	1.42 (1.37–1.47)	< 0.001	1.14 (1.03–1.25)	0.009
Sugammadex	0.45 (0.32–0.61)	< 0.001	0.61 (0.42–0.89)	0.011
BUN > 23 mg/dL	4.08 (2.95–5.64)	< 0.001	1.42 (0.9–2.23)	0.129
Creatinine > 1.2 mg/dL	4.4 (3.08–6.3)	< 0.001	1.44 (0.88–2.37)	0.148
aPTT > 40 s	2.09 (1.66–2.64)	< 0.001	0.97 (0.73–1.29)	0.829
PT, INR > 1.2	4.43 (3.45–5.68)	< 0.001	1.25 (0.92–1.69)	0.156
Platelet < 50,000 μL^−1^	8.04 (4.13–15.66)	< 0.001	1.14 (0.53–2.47)	0.733
Albumin < 3.5 g/dL	10.51 (8.04–13.73)	< 0.001	1.92 (1.35–2.74)	< 0.001
Abdomen surgery	3.45 (1.11–10.76)	0.033	3.27 (0.98–10.86)	0.053
Musculoskeletal surgery	0.77 (0.53–1.1)	0.153	1.49 (0.92–2.4)	0.102
Neurosurgery	3.96 (0.56–28.2)	0.17	38.84 (1.9–794.83)	0.017
OBGY surgery	0 (0–Inf)	0.992	0 (0–Inf)	0.999
Spine surgery	4.63 (0.65–33.03)	0.126	56.71 (1.59–2025.93)	0.027
Thoracic surgery	14.88 (6.57–33.69)	< 0.001	2.27 (0.89–5.81)	0.086
Vascular surgery	0 (0–Inf)	0.991	0 (0–Inf)	0.998
Skin and soft tissue surgery	1.12 (0.53–2.37)	0.767	1.11 (0.41–2.98)	0.843
Major operation surgery	0.51 (0.16–1.59)	0.245	0.12 (0.01–0.93)	0.043
Head and neck (2nd grade/3rd grade)	1.35 (1.23–1.49)	< 0.001	1.06 (0.95–1.2)	0.299
Trunk (2nd grade/3rd grade)	1.37 (1.26–1.5)	< 0.001	1.11 (0.99–1.24)	0.08
Shoulder and upper limb, except wrist and hand (2nd grade/3rd grade)	1.07 (0.98–1.16)	0.134	1.01 (0.89–1.15)	0.845
wrist and hand (2nd grade/3rd grade)	0.96 (0.88–1.04)	0.317	0.95 (0.85–1.05)	0.304
Hip and lower limb, except ankle and foot (2nd grade/3rd grade)	1.22 (1.12–1.32)	< 0.001	1.03 (0.92–1.15)	0.604
ankle and foot (2nd grade/3rd grade)	1.07 (0.97–1.18)	0.186	1.06 (0.94–1.19)	0.34
Eye and adnexa	0.75 (0.24–2.34)	0.622	1.18 (0.3–4.64)	0.811
Respiratory tract	2.3 (1.51–3.48)	< 0.001	1.6 (0.96–2.67)	0.072
Other internal organs	3.03 (0.42–21.56)	0.269	7.74 (0.75–79.61)	0.085
Period from burn to surgery (day)	0.98 (0.97–0.99)	< 0.001	1 (1–1)	0.298
Chemical burn	0.11 (0.05–0.27)	< 0.001	0.66 (0.25–1.72)	0.393
Electrical burn	0.29 (0.21–0.39)	< 0.001	0.86 (0.57–1.3)	0.479
Burn size grade	1.5 (1.44–1.57)	< 0.001	1.13 (1.04–1.23)	0.005
Intraoperative NSIADs	0.71 (0.27–1.92)	0.505	0.73 (0.26–2.06)	0.55
NSAIDs in patient-controlled analgesia	0.63 (0.49–0.82)	< 0.001	1.03 (0.73–1.45)	0.863
Duration of postoperative AAP (day)	1.1 (1.07–1.12)	< 0.001	1.06 (1.03–1.1)	< 0.001
Duration of postoperative Nefopam (day)	0.9 (0.83–0.97)	0.007	1.09 (1.01–1.18)	0.031
Duration of postoperative NSAIDs (day)	0.83 (0.79–0.87)	< 0.001	0.91 (0.87–0.95)	< 0.001

*AAP* acetaminophen, *aPTT* activated partial thromboplastin time, *ASA PS* American Society of Anesthesiologists physical status, BMI body mass index, BUN blood urea nitrogen, *CI* confidence interval, *HR* hazard ratio, *Inf* infinity, *INR* international normalized ratio, *n* number of surgical cases, *NMB* neuromuscular blocker, *NSAID* non-steroidal anti-inflammatory drug, *OBGY* obstetric and gynecological, *PRBC* packed red blood cell, *Preop.* preoperative, *PT* prothrombin time.

Burn size grade: 0 (< 10%)–9 (90–99%).

#### Sensitivity analysis for the risk of postoperative pulmonary complications

A total of 1251 severe burn patients were included in a study to confirm the risk of postoperative pulmonary complications in severe burn patients who underwent major surgery with sugammadex. They underwent 2359 surgeries. Among all patients, postoperative pulmonary complications were observed in 317 cases, accounting for 25.3% of the total patient population and occurring in 13.4% of all surgeries. Sugammadex was used in 681 (28.9%) of the 2359 surgeries. Patient burn characteristics, demographic characteristics of the surgical cases, and preoperative data are summarized in Supplementary Tables 1 and 2, respectively. In the analysis assessing the effect of sugammadex using time-varying Cox regression, the unadjusted HR of sugammadex for the nonuse of sugammadex was 0.46 (95% CI 0.34–0.62; P < 0.001), and the adjusted HR including other variables was 0.62 (95% CI 0.43–0.91; P = 0.013). Supplementary Table 3 summarizes the unadjusted and adjusted HRs of sensitivity analysis.

#### Subgroup analysis for the risk of postoperative pulmonary complications

Of the 859 surgeries (783 patients) performed within 14 days of injury, postoperative pulmonary complications were observed in 274 cases. This represents 35.0% of the 783 patients and 31.9% of the surgeries performed within 14 days of injury. Of the 859 surgeries, 267 (31.1%) were administered sugammadex. Of the 1400 surgeries (753 patients) performed after 14 days of injury, postoperative pulmonary complications were observed in 39 cases. This represents 5.2% of the 753 patients and 2.8% of the surgeries performed after 14 days of injury. Of the surgeries, 267 (31.1%) were administered sugammadex. Of the 1400 surgeries, 382 (27.3%) were administered sugammadex. Burn characteristics of the subgroups are summarized in Supplementary Table 4; their demographic characteristics and perioperative data, in Supplementary Table 5 and 6.

**Figure 3:**
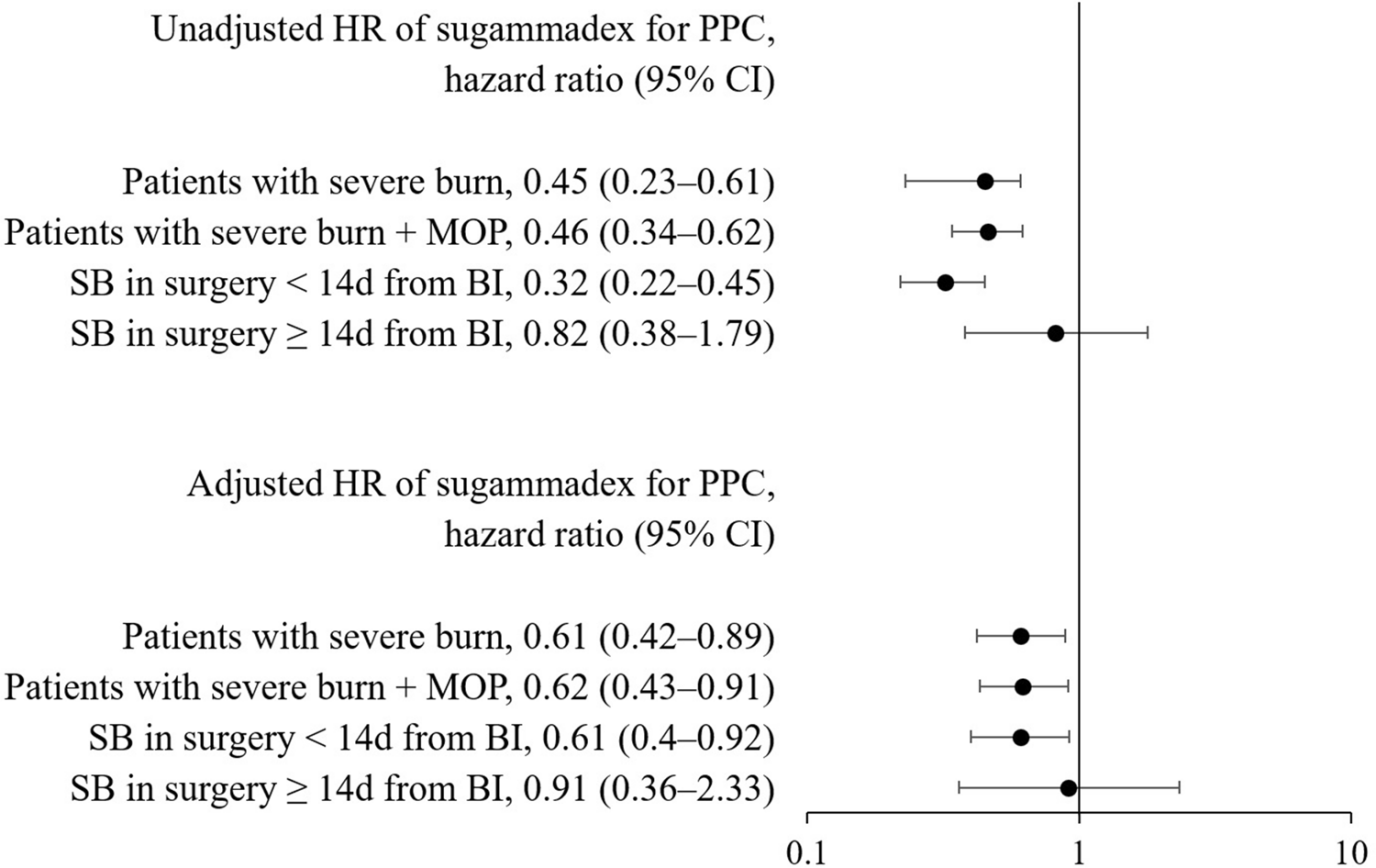
Hazard ratio of sugammadex according to original analysis, sensitivity analysis, and subgroup analysis. *BI* burn injury, *CI* confidence interval, *HR* hazard ratio, *MOP* major operation, *PPC* postoperative pulmonary complication, *SB* severe burn.

In the analysis assessing the effect of sugammadex using time-varying Cox regression, the unadjusted HR of sugammadex for the nonuse of sugammadex were 0.32 (95% CI 0.22–0.45; P < 0.001) for surgery performed within 14 days after burn injury and 0.82 (95% CI 0.38–1.79; P = 0.619) for surgery performed 14 days or more after burn injury. The adjusted HRs for other variables were 0.61 (95% CI 0.4–0.92; P = 0.019) and 0.91 (95% CI 0.36–2.33; P = 0.842), respectively. Unadjusted and adjusted HRs for all the variables with regard to time of surgery are summarized in Supplementary Tables 7 and 8, respectively. Figure 3 depicts HRs for sugammadex according to original analysis, sensitivity analysis and subgroup analysis.

### Type of occurred lung complication and number of undergone surgeries in included each patient

The most common postoperative pulmonary complication in patients with severe burn who underwent surgery was atelectasis, which occurred in 166 cases, representing 13.7% of all patients and 7.3% of all surgeries. The second most common complication was pleural effusion, which occurred in 149 cases, representing 12.3% of all patients and 6.6% of all surgeries. Notably, 58 (18.5%) patients had ≥ 2 complications among patients with postoperative pulmonary complications. Details of postoperative pulmonary complications are summarized in Supplementary Table 9.

### Number of surgeries that patients with severe burn underwent

Of the patients, 465 (38.3%) underwent two or more surgeries during observation periods. Of the patients who underwent surgery within 14 days after the burn injury, only 66 (8.4%) underwent two or more procedures during the observation period. In contrast, the patients who underwent surgery 14 days or more after the burn injury, 297 (39.4%) underwent two or more procedures during the observation period. Details of number of surgeries are summarized in Supplementary Table 10.

### Power calculation

We used the ‘pwr’ library in R to analyze a study of the effect of sugammadex on postoperative complications in patients with severe burn who underwent surgery. The study included 1610 surgical cases which used sugammadex and 649 cases which did not use sugammadex. The event occurrence rate was 16.6% in the sugammadex group and 6.9% in the control group. The power of the study was > 0.999, indicating that it was very likely to detect a statistically significant difference in the event occurrence rate between the two groups.

## Discussion

Our investigation revealed a significant reduction in postoperative pulmonary complications associated with sugammadex use in severe burn patients undergoing surgery under general anesthesia. Notably, our observed incidence of postoperative pulmonary complications, at 25.8%, this rate was not markedly higher than the reported incidence of postoperative pulmonary complications^14,15^. The predominant and overlapped complications observed, including atelectasis and pleural effusion, underscore the complexities and challenges of postoperative pulmonary complications faced in this patient population. Furthermore, our findings indicate that sugammadex administration led to a 39% reduction in the risk of postoperative pulmonary complications compared to nonuse.

Reviewing existing literature reveals a heterogeneous landscape regarding the impact of sugammadex on postoperative pulmonary complications. A multicenter prospective observational study involving 22,803 European patients concluded that choosing sugammadex over neostigmine did not yield superior pulmonary outcomes^16^. However, divergent results emerged from recent large-scale retrospective analyses. Kheterpal et al. reported a substantial 30% reduction in pulmonary complications associated with sugammadex use in a multicenter matched cohort analysis^13^. Conversely, Li et al. contradicted these findings, suggesting no association between neuromuscular blockade reversal agents and postoperative pulmonary complications, citing temporal bias in Kheterpal et al.’s analysis. It’s important to note that Li et al.’s study was conducted within a single center^17^. These conflicting conclusions underscore the inconsistency prevalent in larger retrospective studies regarding sugammadex’s effects on pulmonary outcomes. Beyond these pivotal studies, smaller-scale investigations in specific patient cohorts have highlighted favorable outcomes associated with sugammadex administration. Caron et al. reported lower rates of decreased percutaneous oxygen saturation and critical respiratory events in morbidly obese patients receiving sugammadex compared to neostigmine^12^. Ünal et al. found a reduction in postoperative respiratory complications and related healthcare costs in patients with obstructive sleep apnea upon sugammadex administration^18^. Cheng et al. demonstrated a reduced risk of postoperative pulmonary complications in patients undergoing robotic surgery with sugammadex^19^. However, a series of studies failed to establish a significant difference in the incidence of postoperative pulmonary complications between patients receiving neostigmine and those administered sugammadex ^11,12,20–25^. Notably, to date, no study has specifically examined the association between sugammadex and postoperative pulmonary complications in patients with severe burns.

It is notable that there is a lack of research examining the specific association between sugammadex and postoperative pulmonary complications in patients specifically dealing with severe burns. This absence highlights a critical gap in understanding the potential effects of sugammadex within this unique patient population. The observed discrepancies across various study designs and diverse patient cohorts underscore the intricate nature of comprehending how sugammadex influences pulmonary outcomes. The persistence of conflicting evidence in larger retrospective analyses contrasts with smaller-scale studies that have shown promising outcomes associated with sugammadex use in specific patient groups. These smaller studies have illuminated potential benefits linked to sugammadex administration, particularly in subsets like morbidly obese individuals, patients with obstructive sleep apnea, and those undergoing specific surgical procedures^12,18,19^. This discrepancy between larger and more focused investigations emphasizes the necessity for further focused research within specific patient populations, such as severe burn patients. The need for more targeted investigations becomes paramount in unveiling the nuanced relationship between sugammadex and postoperative pulmonary complications, especially in populations with unique physiological considerations like severe burn patients. As such, future studies should prioritize examining the impact of sugammadex within these specific populations to provide a more comprehensive understanding of its effects on pulmonary outcomes.

Patients with burns undergoing surgery with anesthesia may be at risk for pulmonary complications^26,27^. Burn injury can easily lead to hypovolemic and distributive shock; therefore, perioperative fluid resuscitation is very important. However, fluid resuscitation can lead to fluid overload, which leads to pulmonary lung edema ^28^. Inhalation injury can lead to pulmonary edema and can occlude the airways, leading to atelectasis and pneumonia^29^. Additionally, late pulmonary sequelae due to circumferential burns to the thorax or scar restriction leads to restrictive lung defects with impaired ventilation, with the subsequent development of respiratory failure due to the loss of chest wall elasticity and reduction of effective lung volume^30^. Patients with deep burn wounds typically undergo multiple surgeries requiring general anesthetic management. In the present study, 8.5% of the patients underwent multiple procedures with general anesthesia. Moreover, atelectasis and pleural effusion were most common postoperative pulmonary complications. These were reported as pulmonary complications in patients with burn^31^. Atelectasis may occur because of decreased movement of the chest wall due to eschar, pain, narcotics, and altered mental status. Additionally, atelectasis develops in patients maintaining the same position by increasing pulmonary blood flow of gravitational area^32^. Although pleural effusion is a common postoperative pulmonary complication and may represent minimal clinical significance, patients may complain of dyspnea^33^. Severe diseases, such as congestive heart failure, pneumonia, and pulmonary embolism, are common causes of pleural effusion^34–36^. Severe burn may induce heart failure among those with pleural effusion^37^.

Generally, patients with burns present with increased resistance to nondepolarizing neuromuscular blocking agents. Burn injury causes the upregulation of nicotinic acetylcholine receptors, which is associated with resistance to nondepolarizing neuromuscular blocking agents and increased sensitivity to depolarizing neuromuscular blocking agents^38,39^. Additionally, plasma protein binding of nondepolarizing neuromuscular blocking agents is higher in patients with burns. Martyn et al. reported that patients with burns required five times higher plasma concentrations of d-tubocurarine to obtain a given percentage of twitch depression compared with healthy subjects^40^. Residual neuromuscular blockade may develop because of changes in neuromuscular function in patients with burns. The residual neuromuscular blockade increases the incidence of upper airway obstruction^41^, which can further lead to pulmonary edema^42^ and prolonged postoperative weaning from mechanical ventilation^43^. This also increases the risk of postoperative pulmonary complications, such as atelectasis and pneumonia^44^. The reduction in the risk of postoperative pulmonary complications was significantly associated with the use of sugammadex in this study. However, approximately 1 week after the occurrence of burns involving > 30% of total body surface area, resistance to all nondepolarizing neuromuscular blockers develops proportional to burn size, with peak resistance occurring 5–6 weeks after injury. Several months to years are necessary to recover to the pre-burn neuromuscular function state after burn injury^45^. In the patients who underwent surgery after 14 days or more of the burn injury, the dose of rocuronium differed according to whether sugammadex was administered: it was higher when sugammadex was administered (median rocuronium dose: 100 mg) than when sugammadex was not administered (median rocuronium dose: 50 mg). Although the development of postoperative pulmonary complication may be associated with a high dose of rocuronium, the rate of postoperative pulmonary complication in our study was higher among patients who underwent early surgery than among those who underwent late surgery. It is uncertain whether included patients had resistance to nondepolarizing neuromuscular blocking agents and whether sugammadex is effective to patients with resistance for neuromuscular blocking agents in the present study.

This study exhibits several notable strengths. Firstly, it pioneers the investigation into the relationship between sugammadex usage and postoperative pulmonary complications, specifically focusing on severe burn patients. This fills a crucial void within the existing literature by addressing a previously unexplored area. Secondly, our research encompasses a substantial volume of surgeries and patients, bolstering the credibility and relevance of our findings within the realm of severe burn patients undergoing surgery. Thirdly, our study delves deeply into the examination of various types and frequencies of postoperative pulmonary complications observed in this specific patient cohort. Lastly, our research conducts a critical appraisal of the current body of literature, drawing comparisons and contrasts. It underscores the imperative need for more tailored investigations aimed at specific patient populations, such as those with severe burn injuries.

However, this study also has several limitations. First, neuromuscular monitoring data was insufficient. Because anesthesia records were stored in an image format, some data that were not stored in numbers were excluded. Although neuromuscular monitoring is important for the selection and use of neuromuscular reversal agents, continuous monitoring data, such as those on neuromuscular monitoring, were recorded in the form of images. In addition, patients had ≥ 50% burns of 2nd or 3rd degree in upper extremities. Because only acceleromyograph monitoring of thumb was used for neuromuscular monitoring in our institutions, many patients’ neuromuscular conditions would not have been able to be monitored. Given that patients with burns exhibit increased resistance to nondepolarizing neuromuscular agents, the residual neuromuscular effect of burn injury should be examined in future studies. Second, some patients who used sugammadex may have used also cholinesterase inhibitors. When sugammadex and cholinesterase inhibitors are used together, only the expensive drug sugammadex can be included in the prescribing data for claiming medical insurance fee because medical insurance covers the cost of only one of the two drugs. Therefore, this study may not be a complete only comparison of sugammadex and cholinesterase inhibitors. Third, the choice of patients receiving sugammadex might be affected by the cost of sugammadex, which is considerably higher than that of neostigmine and pyridostigmine, and the difference of medication costs might have impacted the patient selection. In particular, sugammadex might have been preferred in patients with a higher risk of postoperative pulmonary complications.

In conclusion, in the present retrospective study, sugammadex significantly reduced the risk of postoperative pulmonary complications in patients who underwent surgery for severe burns, and we reported clinically relevant information on the selection of sugammadex as a neuromuscular blockade reversal agent to reduce the incidence of postoperative pulmonary complications. However, because this study has some limitations, future randomized controlled trials involving neuromuscular monitoring data of patients with burn are warranted to confirm these findings.

## Methods

### Ethical approval/informed consent

This retrospective study was conducted in the Hallym University Medical Center in Korea and was approved by the Clinical Research Ethics Committee of the Chuncheon Sacred Heart Hospital, Hallym University (Approval No. 2021-06-017). The study included fragile participants, such as critical ill patients. Because it was a retrospective analysis of clinical data acquired after the treatment process, the institution waived the requirement of informed consent of all participants.

### Data sources, study design and setting

All data were obtained from the clinical data warehouse (CDW) of five hospitals (Chuncheon Sacred Heart Hospital, Dongtan Sacred Heart Hospital, Hallym University Sacred Heart Hospital, Hangang Sacred Heart Hospital and Kangnam Sacred Heart Hospital) of the Hallym University Medical Centre (capacity of 3100 beds). The clinical data warehouse is a database of medical records, prescriptions, and test results from the Hallym University Medical Centre spanning around 11 years starting in 1 January 2011. As sugammadex has been used since 2013, our study only included data since 2013. We conducted this retrospective cohort study from January 2013 to November 2021. This manuscript adheres to the applicable STROBE guidelines.

### Participants and burns

The present study included adult patients (≥ 18 years) with severe burns who underwent surgeries under general anesthesia. The diagnosis of burn was made according to the Korean Standard Classification of Disease system, which is derived from and is similar to the International Classification of Diseases system. The diagnostic codes of burns used in the present study are summarized in Supplementary Table 11. We defined the following burns as severe: burn complicated by inhalation injury, chemical burn, and high-voltage electrical burn; moreover, in adults, any burn encompassing ≥ 20% of the total body surface area, excluding superficial burns (first-degree burns) was classified as severe. For older adults, burns were defined as severe when they encompassed < 20% (10–19%) of the total body surface area^46,47^. Older adult was defined 70 and more years old in this study. The exclusion criteria were as follows: patients with preoperative pulmonary complications, those with missing medical records, those who did not use steroidal neuromuscular blocking drugs, those who did not use cholinesterase inhibitors or sugammadex, and those with outlier records. Cholinesterase inhibitors included neostigmine and pyridostigmine. When patients had additional burn injury, surgery cases after additional burn injury were not included for analysis.

### Primary outcomes and secondary outcomes

In the present study, the primary outcome was postoperative pulmonary complications, which included atelectasis, pulmonary edema, pulmonary effusion, pneumothorax, pneumonia, pulmonary thromboembolism, respiratory failure and acute respiratory distress. Supplementary Table 12 lists the diagnostic approaches used for pulmonary complications. All radiological image readings were performed by board certification of the radiologists. If the patient had not been tested for postoperative pulmonary complication diagnosis, the patient was determined to be postoperative pulmonary complication-free. Secondary outcomes are type of occurred lung complication and frequency of undergone surgeries in included each patient.

### Observation period

The observation period for each patient commenced at the conclusion of their initial surgery. This period extended from the completion of the first surgery until the commencement of subsequent surgeries or the manifestation of postoperative pulmonary complications or discharged. In instances where patients underwent multiple surgeries, each sequential observation time (*n*-th observation time) was delineated from the conclusion of the *n*-th surgery until the initiation of the subsequent surgery or the occurrence of postoperative pulmonary complications following the surgery or discharged.

### Exposure variable and covariates

The exposure variable was administration of sugammadex. The other study variables were the characteristics of burn and patient data, and perioperative data. The burn characteristics included area, grade, and type of burn (fire, chemical, or electrical), which are summarized in Supplementary Table 13.

The demographic characteristics of patients were documented before every surgical procedure, and perioperative data were measured before and during every operation. The demographic characteristics included old age (≥ 70 years), sex, obesity (body mass index ≥ 30 kg/m^2^), preoperative coexisting disease (e.g., congestive heart failure, cardiac arrhythmias, valvular disease, pulmonary circulation disorders, peripheral vascular disorders, uncomplicated hypertension, complicated hypertension, paralysis, other neurological disorders, chronic pulmonary disease, uncomplicated diabetes, complicated diabetes, hypothyroidism, renal failure, liver disease, peptic ulcer disease excluding bleeding, coagulopathy, fluid and electrolyte disorders, deficiency anemia, alcohol abuse, drug abuse, psychosis, depression), alcohol and smoking. The perioperative data included emergency status, American Society of Anesthesiologists physical status of > 2, N_2_O use, inhalation anesthetics, rocuronium, arterial line monitoring, central venous line monitoring, Foley catheter use, patient controlled analgesia, blood urea nitrogen > 23 mg/dL, creatinine > 1.2 mg/dL, activated partial thromboplastin time > 40 s, prothrombin time (international normalized ratio > 1.2), platelet count < 50,000 µL^−1^, albumin < 3.5 g/dL, abdominal surgery, musculoskeletal surgery, neurosurgery, obstetric and gynecological surgery, spinal surgery, thoracic surgery, vascular surgery, skin and soft tissue surgery, major surgery, surgery time, intraoperative fluid balance, estimated intraoperative blood loss, intraoperative packed red blood cell transfusion, and the period between the burn and the surgery. The definition of unfixed repetitive variables was summarized in Supplementary Table 14.

### Data handling and extraction

All variables utilized in the study were meticulously defined and structured in Supplementary Tables 12–14. Data extraction involved accessing the Clinical Data Warehouse (CDW) integrated software, encompassing patient characteristics, laboratory results, imaging tests, diagnostic codes, and prescription information. The CDW facilitated the extraction and organization of pertinent data.

The specifics related to burn site and extent were categorized using diagnostic codes, while records of chemical and electrical burns were extracted from burn assessment records and subsequently categorized. Surgical types and comorbidities were designated using surgical codes and diagnostic codes as covariates. Laboratory test results were stratified from normal values. Other medications or treatments were categorized and extracted from prescription records. Additional patient characteristics were gathered from the electronic medical records.

Continuous variables were categorized based on predefined criteria, outlined comprehensively in Supplementary Tables 12–14. Variables such as burn site and size were treated as ordinal variables aligned with burn grade, while other binary variables were transformed using one-hot encoding.

### Statistical methods

Continuous variables were expressed as medians with interquartile ranges, and categorical variables were expressed as frequencies with percentages. Because continuous data were skewed, they were analyzed using the Mann–Whitney test. Categorical data were analyzed using the chi-square test or Fisher’s exact test. To evaluate the difference in incidence of postoperative pulmonary complications according to sugammadex administration in each surgical case, Kaplan–Meier and Log rank test was used.

In our study, various statistical measures were employed to account for potential confounding factors that could impact the interpretation of our results. To address confounding, we employed several statistical methods.

#### Covariate adjustment

Covariates such as age, sex, obesity, preoperative coexisting diseases, perioperative variables (emergency status, anesthesiologists’ physical status, specific surgical procedures, surgical timing, intraoperative factors), and other pertinent variables were included in our analyses. These were adjusted for in regression models to mitigate their potential confounding effects on the association between sugammadex administration and postoperative pulmonary complications.

#### Time-varying covariables in Cox proportional hazards model

As severe burn patients often undergo multiple surgeries, we used the Cox proportional hazards model with time-varying covariables. This method can help in adjusting for the changing effects of these variables as they evolve across different surgical interventions or stages of patient treatment. In our study on severe burn patients who might undergo multiple surgeries, using time-varying covariables in the Cox model allowed to consider the dynamic nature of the exposure (sugammadex administration) and other covariates over time to estimate hazard ratios (HRs) and 95% confidence intervals (CIs) while adjusting for potential confounders. By accounting for these changes over time, the Cox proportional hazards model with time-varying covariables can provide more accurate and precise estimates of the hazard ratios and survival probabilities associated with sugammadex administration while considering potential confounding factors that may vary over the course of patient treatment.

#### Sensitivity analysis

We conducted sensitivity analyses by including patients who underwent major surgery for burn injury in the previously defined severe burn patient group. Because severe burn may be defined as a burn complicated by major trauma^47^, we defined major trauma as major surgery for injury, and performed sensitivity analysis by who underwent major surgery for burn injury.

Subgroup analysis: It is widely known that early surgery is better than delayed surgery, and surgeons preferred early surgery^48^. Early excision and grafting of the burn, noted on days 2–12 after the burn, is the most important operation during the patient’s hospitalization. For cosmetic areas and functional areas, excision should be performed at least within 7 to 14 days to improve results^7^. Skin grafts that were performed within 14 days of the injury improve scar outcome^49^. Therefore, early surgery is generally performed and preferred in patients with burn. We defined early surgery as the surgery being performed within 14 days after burn injury, through few reports^6,49^. The patients were categorized into two subgroups according to whether they underwent early surgery or surgery 14 days or more after burn injury. We investigated effects of sugammadex for developing postoperative pulmonary complication in two subgroups.

The Google Colab (Python version 3.7; Mountain View, CA, USA) and R version 4.2.3 (R Foundation for Statistical Computing, www.r-project.org [accessed on 21 November 2023]) was used for all statistical analyses. Power calculation was conducted using R version 4.2.3. P-values of < 0.05 were considered statistically significant.

### Supplementary Information

Supplementary Tables.

## Data Availability

The datasets used and analyzed during the current study are available from the corresponding author on reasonable request.
