# Supplementary material for: Effect of sugammadex on postoperative complications in patients with severe burn who underwent surgery: a retrospective study
Source: Sci Rep. 2024 Jan 4;14:525. doi: 10.1038/s41598-024-51171-y (PMC10767056; doi:10.1038/s41598-024-51171-y)
Supplement: Supplementary file 1 — Supplementary Tables. [file 41598_2024_51171_MOESM1_ESM.pdf]

Effect of sugammadex on postoperative complications in patients with severe burn who underwent surgery: a retrospective study

Jong Ho Kim, MD[1,2]; Minguan Kim MD[1]; Minho Oh[3], Soo-Kyung Lee[3]; Young Suk Kwon, MD[1,2,*]

Supplementary table 1. Burn characteristics in patients with severe burn who underwent surgery or those with major surgery.

Characteristics of burn	No PPC (n = 934)	PPC (n =317)	P value
Head and neck (2nd grade / 3rd grade)	233 (24.9) / 47 (5.0)	117 (36.9) / 37 (11.7)	<0.001†
Trunk (2nd grade / 3rd grade)	167 (17.9) / 125 (13.4)	83 (26.2) / 86 (27.1)	<0.001†
Shoulder and upper limb, except wrist and hand (2nd grade / 3rd grade)	166 (17.8) / 167 (17.9)	48 (15.1) / 77 (24.3)	0.002†
Wrist and hand (2nd grade / 3rd grade)	207 (22.2) / 270 (28.9)	65 (20.5) / 91 (28.7)	0.705†
Hip and lower limb, except ankle and foot (2nd grade / 3rd grade)	175 (18.7) / 153 (16.4)	40 (12.6) / 105 (33.1)	<0.001†
Ankle and foot (2nd grade / 3rd grade)	77 (8.2) / 120 (12.8)	19 (6.0) / 54 (17.0)	0.028†
Eye and adnexa	13 (1.4)	3 (0.9)	0.772*
Respiratory tract	28 (3.0)	24 (7.6)	<0.001†
Other internal organs	1 (0.1)	1 (0.3)	0.443*
Chemical burn	142 (15.2)	5 (1.6)	<0.001*
Electrical burn	387 (41.4)	53 (16.7)	<0.001†
Burn size grade	1.0 (0.0, 2.0)	3.0 (2.0, 5.0)	<0.001†

Data are presented as numbers (%) or medians (interquartile range)

Burn size grade: 0, <10 % of TBS; 1, 10–19% of TBS; 2, 20–29% of TBS; 3, 30–39% of TBS; 5, 50–59% of TBS

PPC, postoperative pulmonary complication; TBS, total body surface

†, chi-square test; *, Fisher's exact test

Supplementary table 2. Demographic characteristics and perioperative data in surgical cases of patients with severe burn who underwent surgery or those with major surgery.

	Nonuse of sugammadex (n = 1678)	Sugammadex (n =681)	P value
Old Age (≥70 years)	141 (8.4)	67 (9.8)	0.339 ‡
Male	1419 (84.6)	575 (84.4)	0.895 ‡
Obesity (BMI ≥30)	53 (3.2)	30 (4.4)	0.162 ‡
Congestive heart failure	16 (0.9)	1 (0.1)	0.055 *
Cardiac arrhythmias	16 (0.9)	12 (1.8)	0.146 ‡
Valvular disease	7 (0.4)	2 (0.3)	>0.999 *
Pulmonary circulation Disorders	2 (0.1)	2 (0.3)	0.326 *
Peripheral vascular disorders	8 (0.5)	0 (0)	0.115 *
Hypertension, uncomplicated	147 (8.8)	50 (7.3)	0.494 ‡
Hypertension, complicated	47 (2.8)	7 (1.0)	0.013 *
Paralysis	8 (0.5)	6 (0.9)	0.245 *
Other neurological disorders	8 (0.5)	4 (0.6)	0.752 *
Chronic pulmonary disease	12 (0.7)	1 (0.1)	0.126 *
Diabetes, uncomplicated	32 (1.9)	18 (2.6)	0.232 ‡
Diabetes, complicated	352 (21.0)	60 (8.8)	<0.001 ‡
Hypothyroidism	3 (0.2)	0 (0)	0.562 *
Renal failure	13 (0.8)	3 (0.4)	0.768 *
Liver disease	46 (2.7)	23 (3.4)	0.537 ‡
Peptic ulcer disease excluding bleeding	10 (0.6)	1 (0.1)	0.461 *
Coagulopathy	4 (0.2)	1 (0.1)	>0.999 *
Fluid and electrolyte disorders	4 (0.2)	0 (0)	>0.999 *
Deficiency anemia	3 (0.2)	2 (0.3)	>0.999 *
Alcohol abuse	1 (0.1)	1 (0.1)	0.492 *
Drug abuse	9 (0.5)	4 (0.6)	>0.999 *
Psychoses	9 (0.5)	6 (0.9)	0.39 *
Depression	227 (13.5)	101 (14.8)	0.237 ‡
Alcohol	881 (52.5)	370 (54.3)	0.358 ‡
Smoking	656 (39.1)	259 (38.0)	0.72 ‡
Emergency	100 (6.0)	49 (7.2)	0.243 ‡
ASA PS > 2	272 (16.2)	105 (15.4)	0.559 ‡
N_2O	1577 (94.0)	656 (96.3)	0.026 ‡
Inhalation anesthetics	1601 (95.4)	675 (99.1)	<0.001 *
Rocuronium, mg	100.0 (50.0, 100.0)	100.0 (100.0, 100.0)	<0.001 †
Surgery time, hour	1.3 (0.8, 2.1)	1.2 (0.8, 1.8)	0.195 †
Intraoperative fluid, L	0.8 (0.4, 1.6)	0.6 (0.3, 1.1)	<0.001 †
Estimated blood loss, L	0.3 (0.1, 0.6)	0.1 (0.0, 0.3)	<0.001 †
Arterial line monitoring	735 (43.8)	299 (43.9)	0.754 ‡
Central venous line monitoring	19 (1.1)	2 (0.3)	0.033 *
Foley catheter	811 (48.3)	308 (45.2)	0.081 ‡
Patient controlled analgesia	1338 (79.7)	588 (86.3)	<0.001 ‡
Intraoperative PRBC, unit	0.0 (0.0, 3.0)	0.0 (0.0, 2.0)	0.02 †
BUN > 23mg·dl^{-1}	64 (3.8)	21 (3.1)	0.408 ‡
Creatinine > 1.2mg·dl^{-1}	59 (3.5)	20 (2.9)	0.399 ‡
aPTT > 40 seconds	375 (22.4)	211 (31.0)	<0.001 ‡
PT, INR > 1.2	178 (10.6)	30 (4.4)	<0.001 ‡
Platelet <50000·$\mu\ell^{-1}$	12 (0.7)	0 (0)	0.024 *
Albumin < 3.5g·dl^{-1}	83 (5.0)	9 (1.3)	<0.001 *
Abdomen surgery	8 (0.5)	1 (0.1)	0.452 *
Musculoskeletal surgery	296 (17.6)	132 (19.4)	0.158 ‡
Neurosurgery	2 (0.1)	3 (0.4)	0.146 *
OBGY surgery	1 (0.1)	0 (0)	>0.999 *
Spine surgery	1 (0.1)	2 (0.3)	>0.999 *

Thoracic surgery	6 (0.4)	0 (0)	0.191 *
Vascular surgery	2 (0.1)	1 (0.1)	>0.999 *
Skin & soft tissue surgery	1607 (95.8)	655 (96.2)	0.668 ‡
Major operation surgery	52 (3.1)	39 (5.7)	0.189 ‡
Period from burn to surgery, day	19.0 (7.0, 46.0)	16.0 (8.0, 37.0)	0.105 †
Intraoperative NSIADs	42 (2.5)	6 (0.9)	0.011‡
NSAIDs in patient controlled analgesia	641 (38.2)	63 (9.3)	<0.001‡
Duration of postop. AAP, day	1 (0, 3)	0 (0, 2)	0.008†
Duration of postop. Nefopam, day	0 (0, 0)	0 (0, 0)	0.029†
Duration of postop. NSAIDs, day	1 (0, 4)	2 (0, 6)	0.104†
Start date, day	0.0 (0.0, 25.0)	6.0 (0.0, 25.0)	0.017 †
Stop date, day	21.9 (11.8, 52.5)	24.0 (13.1, 46.9)	0.172 †

Data are presented as numbers (%) or medians (interquartile range)

AAP, acetaminophen; aPTT, activated partial thromboplastin time; ASA PS, American Society of Anesthesiologists physical status; body mass index; BUN, blood urea nitrogen; INR, international normalized ratio; n, number of surgical cases; NSAID, non-steroidal anti-inflammatory drug; OBGY, obstetric and gynecological; PRBC, packed red blood cell; PT, prothrombin time

† Start date, start of observation based on the date of first surgery (observation start date – date of first surgery);

†† Stop date, end of observation based on the date of first surgery (observation stop date – date of first surgery)

‡, chi-square test; *, Fisher's exact test; ‖, Mann-Whitney test

Supplementary table 3. Unadjusted and adjusted hazard ratio on developing postoperative pulmonary complications in patients with severe burn who underwent or those with major surgery.

	Unadjusted HR (95% CI)	P value	Adjusted HR (95% CI)	P value
Old Age (≥70 years)	2.1 (1.6–2.76)	<0.001	1.91 (1.29–2.83)	0.001
Male	0.93 (0.7–1.22)	0.587	0.96 (0.68–1.36)	0.864
Obesity (BMI≥30)	1.68 (1.02–2.78)	0.044	1.23 (0.71–2.12)	0.466
Congestive heart failure	2.82 (1.17–6.83)	0.021	1.31 (0.43–3.98)	0.686
Cardiac arrhythmias	2.55 (1.26–5.14)	0.009	1.6 (0.57–4.46)	0.365
Valvular disease	1.92 (0.48–7.71)	0.359	1.08 (0.15–7.56)	0.931
Pulmonary circulation Disorders	2.3 (0.57–9.22)	0.242	16.65 (2.17–127.76)	0.007
Peripheral vascular disorders	0.83 (0.12–5.93)	0.854	9.69 (1.13–83.35)	0.039
Hypertension, uncomplicated	1.26 (0.88–1.82)	0.212	0.9 (0.52–1.56)	0.732
Hypertension, complicated	1.42 (0.75–2.66)	0.28	1.34 (0.61–2.95)	0.472
Paralysis	1.98 (0.64–6.18)	0.238	0.43 (0.1–1.97)	0.281
Other neurological disorders	4.31 (1.38–13.46)	0.012	3.42 (0.94–12.52)	0.062
Chronic pulmonary disease	0 (0–Inf)	0.991	0 (0–Inf)	0.991
Diabetes, uncomplicated	0.96 (0.43–2.14)	0.912	1.21 (0.42–3.48)	0.732
Diabetes, complicated	0.9 (0.66–1.22)	0.486	1.03 (0.71–1.49)	0.906
Hypothyroidism	2.8 (0.39–19.98)	0.303	3.47 (0.43–27.71)	0.24
Renal failure	2.8 (1.25–6.29)	0.012	1.44 (0.49–4.25)	0.424
Liver disease	1.16 (0.57–2.33)	0.687	1.86 (0.87–3.97)	0.111
Peptic ulcer disease excluding bleeding	0 (0–Inf)	0.991	0 (0–Inf)	0.993
Coagulopathy	7.1 (2.64–19.14)	<0.001	0.67 (0.22–2.06)	0.473
Fluid and electrolyte disorders	1.71 (0.24–12.15)	0.594	0.23 (0.01–5.05)	0.352
Deficiency anemia	1.93 (0.48–7.76)	0.354	1.72 (0.27–10.97)	0.55
Alcohol abuse	0 (0–Inf)	0.989	0 (0–Inf)	0.997
Drug abuse	1.75 (0.56–5.45)	0.335	1.3 (0.37–4.63)	0.746
Psychoses	3.16 (1.18–8.48)	0.022	2.1 (0.73–6.07)	0.168
Depression	0.93 (0.65–1.32)	0.67	0.78 (0.52–1.18)	0.252
Alcohol	0.95 (0.76–1.19)	0.654	1.11 (0.84–1.46)	0.46
Smoking	1.05 (0.84–1.32)	0.669	0.96 (0.73–1.27)	0.789
Emergency	2.99 (2.26–3.96)	<0.001	1.44 (1.03–2.02)	0.034

ASA PS > 2	7.01 (5.57–8.81)	<0.001	1.65 (1.22–2.22)	0.001
N2O	0.28 (0.21–0.37)	<0.001	0.65 (0.45–0.93)	0.016
Inhalation anesthetics	2.52 (1.04–6.09)	0.041	1.28 (0.48–3.4)	0.627
Rocuronium	1.01 (1–1.01)	<0.001	1 (1–1)	0.566
Surgery time, hour	1.11 (1.03–1.2)	0.004	1.02 (0.91–1.13)	0.676
Fluid, Liter	1.26 (1.22–1.3)	<0.001	1.08 (0.96–1.21)	0.189
Estimated blood loss, Liter	2.47 (2.2–2.76)	<0.001	0.78 (0.57–1.08)	0.141
Arterial line monitoring	13.23 (8.72–20.08)	<0.001	3.27 (1.9–5.61)	<0.001
Central venous line monitoring	2.49 (1.23–5.02)	0.011	1.14 (0.53–2.47)	0.639
Foley catheter	11.56 (7.62–17.53)	<0.001	1.89 (1.12–3.19)	0.016
Patient-controlled analgesia	0.32 (0.26–0.41)	<0.001	0.81 (0.59–1.1)	0.183
Intraoperative PRBC, unit	1.38 (1.34–1.43)	<0.001	1.1 (1.01–1.21)	0.042
Sugammadex	0.46 (0.34–0.62)	<0.001	0.62 (0.43–0.91)	0.013
BUN > 23mg·dl^{-1}	4.01 (2.9–5.55)	<0.001	1.42 (0.9–2.23)	0.138
Creatinine > 1.2mg·dl^{-1}	4.03 (2.82–5.76)	<0.001	1.34 (0.82–2.2)	0.276
aPTT > 40 seconds	2.04 (1.62–2.56)	<0.001	0.97 (0.73–1.28)	0.848
PT, INR > 1.2	4.41 (3.44–5.65)	<0.001	1.26 (0.94–1.7)	0.115
Platelet <50000·$\mu\ell^{-1}$	8.32 (4.27–16.2)	<0.001	1.18 (0.55–2.53)	0.661
Albumin < 3.5g·dl^{-1}	10.88 (8.32–14.22)	<0.001	1.94 (1.36–2.77)	<0.001
Abdomen surgery	3.3 (1.06–10.29)	0.04	3.2 (0.97–10.59)	0.057
Musculoskeletal surgery	0.76 (0.53–1.09)	0.136	1.41 (0.88–2.24)	0.157
Neurosurgery	4.1 (0.58–29.21)	0.159	21.89 (1.64–293.06)	0.021
OBGY surgery	0 (0–Inf)	0.992	0 (0–Inf)	0.999
Spine surgery	2.8 (0.7–11.25)	0.147	25.41 (2.4–268.57)	0.008
Thoracic surgery	15.38 (6.79–34.84)	<0.001	2.24 (0.88–5.73)	0.087
Vascular surgery	0 (0–Inf)	0.992	0 (0–Inf)	0.998
Skin & soft tissue surgery	0.95 (0.49–1.84)	0.874	0.96 (0.38–2.41)	0.929
Major operation surgery	0.42 (0.19–0.94)	0.036	0.16 (0.04–0.59)	0.006
head and neck (2nd grade / 3rd grade)	1.36 (1.23–1.49)	<0.001	1.05 (0.93–1.17)	0.487
trunk (2nd grade / 3rd grade)	1.39 (1.28–1.51)	<0.001	1.11 (0.99–1.24)	0.08
shoulder and upper limb, except wrist and hand (2nd grade / 3rd grade)	1.08 (0.99–1.18)	0.082	1.02 (0.9–1.15)	0.747
wrist and hand (2nd grade / 3rd grade)	0.96 (0.89–1.05)	0.358	0.96 (0.86–1.06)	0.392
hip and lower limb, except ankle and foot (2nd grade / 3rd grade)	1.22 (1.13–1.33)	<0.001	1.01 (0.91–1.12)	0.846

ankle and foot (2nd grade / 3rd grade)	1.05 (0.95–1.16)	0.325	1.04 (0.93–1.17)	0.489
eye and adnexa	0.77 (0.25–2.41)	0.657	1.28 (0.33–4.88)	0.729
respiratory tract	2.36 (1.56–3.58)	<0.001	1.61 (0.97–2.67)	0.065
other internal organs	3.13 (0.44–22.32)	0.254	6.25 (0.64–60.62)	0.114
Period from burn to surgery, day	0.99 (0.98–0.99)	<0.001	1 (1–1)	0.409
Chemical burn	0.12 (0.05–0.28)	<0.001	0.7 (0.27–1.8)	0.449
Electrical burn	0.3 (0.22–0.4)	<0.001	0.86 (0.58–1.28)	0.434
Burn size grade	1.51 (1.44–1.57)	<0.001	1.14 (1.05–1.24)	0.002
Intraoperative NSIADs	0.73 (0.27–1.97)	0.537	0.7 (0.25–1.98)	0.511
NSAIDs in patient-controlled analgesia	0.63 (0.48–0.82)	<0.001	1.04 (0.74–1.46)	0.997
Duration of postoperative AAP, day	1.09 (1.06–1.11)	<0.001	1.07 (1.03–1.1)	0.843
Duration of postoperative Nefopam, day	0.87 (0.81–0.94)	<0.001	1.06 (0.98–1.15)	<0.001
Duration of postoperative NSAIDs, day	0.87 (0.84–0.91)	<0.001	0.95 (0.91–0.99)	0.162

AAP, acetaminophen; aPTT, activated partial thromboplastin time; ASA PS, American Society of Anesthesiologists physical status; BMI, body mass index; BUN, blood urea nitrogen; CI, confidence interval; HR, hazard ratio; Inf, infinity; INR, international normalized ratio; n, number of surgical cases; NMB, neuromuscular blocker; NSAID, non-steroidal anti-inflammatory drug; OBGY, obstetric and gynecological; PRBC, packed red blood cell; Preop., preoperative; PT, prothrombin time; TBS, total body surface

Burn size grade: 0 (<10%) ~ 9 (90–99%)

Supplementary table 4. Burn characteristics in patients with severe burn who underwent surgery within 14 days from burn injury and those at 14 and more days from burn injury.

Characteristics of burn	< 14 days form burn (n = 783)			≥14 days from burn (n = 753)		
	**No PPC (n = 509)**	**PPC (n =274)**	**P value**	**No PPC (n = 714)**	**PPC (n =39)**	**P value**
Head and neck (2nd grade / 3rd grade)	148 (29.1) / 28 (5.5)	106 (38.7) / 33 (12.0)	<0.001‡	166 (23.2) / 39 (5.5)	11 (28.2) / 4 (10.3)	0.149‡
Trunk (2nd grade / 3rd grade)	106 (20.8) / 80 (15.7)	73 (26.6) / 82 (29.9)	<0.001‡	127 (17.8) / 99 (13.9)	10 (25.6) / 3 (7.7)	0.305‡
Shoulder and upper limb, except wrist and hand (2nd grade / 3rd grade)	90 (17.7) / 116 (22.8)	43 (15.7) / 73 (26.6)	0.446‡	125 (17.5) / 121 (16.9)	4 (10.3) / 4 (10.3)	0.22‡
Wrist and hand (2nd grade / 3rd grade)	112 (22.0) / 174 (34.2)	58 (21.2) / 81 (29.6)	0.39‡	158 (22.1) / 219 (30.7)	7 (17.9) / 10 (25.6)	0.769‡
Hip and lower limb, except ankle and foot (2nd grade / 3rd grade)	105 (20.6) / 103 (20.2)	35 (12.8) / 92 (33.6)	<0.001‡	132 (18.5) / 115 (16.1)	5 (12.8) / 13 (33.3)	0.004‡
Ankle and foot (2nd grade / 3rd grade)	45 (8.8) / 73 (14.3)	17 (6.2) / 45 (16.4)	0.353‡	53 (7.4) / 85 (11.9)	2 (5.1) / 7 (17.9)	0.293‡
Eye and adnexa	6 (1.2)	3 (1.1)	>0.999*	10 (1.4)	0 (0)	>0.999
Respiratory tract	13 (2.5)	24 (8.8)	<0.001‡	22 (3.1)	0 (0)	0.62*
Other internal organs	1 (0.2)	1 (0.4)	>0.999*	0 (0)	0 (0)	
Chemical burn	68 (13.4)	4 (1.5)	<0.001*	90 (12.6)	1 (2.6)	0.074*
Electrical burn	226 (44.4)	42 (15.3)	<0.001‡	332 (46.5)	11 (28.2)	0.039‡
Burn size grade	1.0 (0.0, 2.0)	3.0 (2.0, 5.0)	<0.001‡	1.0 (0.0, 2.0)	2.0 (1.0, 3.0)	0.057‡

Data are presented as numbers (%) or medians (interquartile range)

Burn size grade: 0, <10 % of TBS; 1, 10–19% of TBS; 2, 20–29% of TBS; 3, 30–39% of TBS; 5, 50–59% of TBS

PPC, postoperative pulmonary complication; TBS, total body surface

‡, chi-square test; *, Fisher's exact test

Supplementary table 5. Demographic characteristics and perioperative data in surgical cases of patients with severe burn who underwent surgery within 14 days from burn injury

	**Nonuse of sugammadex (n = 592)**	**Sugammadex (n =267)**	**P value**
Old Age (≥70 years)	89 (15.0)	34 (12.7)	0.432‡
Male	501 (84.6)	213 (79.8)	0.097‡
Obesity (BMI≥30)	26 (4.4)	9 (3.4)	0.578*
Congestive heart failure	7 (1.2)	0 (0)	0.106*
Cardiac arrhythmias	8 (1.4)	4 (1.5)	>0.999*
Valvular disease	4 (0.7)	1 (0.4)	>0.999*
Pulmonary circulation Disorders	1 (0.2)	1 (0.4)	0.525*
Peripheral vascular disorders	1 (0.2)	0 (0)	>0.999*
Hypertension, uncomplicated	54 (9.1)	14 (5.2)	0.07‡
Hypertension, complicated	14 (2.4)	2 (0.8)	0.17*
Paralysis	4 (0.7)	4 (1.5)	0.263*
Other neurological disorders	3 (0.5)	3 (1.1)	0.382*
Chronic pulmonary disease	2 (0.3)	1 (0.4)	>0.999*
Diabetes, uncomplicated	14 (2.4)	6 (2.2)	>0.999*
Diabetes, complicated	105 (17.7)	18 (6.7)	<0.001‡
Hypothyroidism	2 (0.3)	0 (0)	>0.999*
Renal failure	5 (0.8)	0 (0)	0.332*
Liver disease	18 (3.0)	5 (1.9)	0.372*
Peptic ulcer disease excluding bleeding	2 (0.3)	1 (0.4)	>0.999*
Coagulopathy	4 (0.7)	1 (0.4)	>0.999*
Fluid and electrolyte disorders	1 (0.2)	0 (0)	>0.999*
Deficiency anemia	1 (0.2)	1 (0.4)	0.525*
Alcohol abuse	0 (0)	1 (0.4)	0.311*
Drug abuse	5 (0.8)	2 (0.8)	>0.999*
Psychoses	3 (0.5)	2 (0.8)	0.649*
Depression	78 (13.2)	35 (13.1)	>0.999‡
Alcohol	325 (54.9)	146 (54.7)	>0.999‡
Smoking	239 (40.4)	110 (41.2)	0.878‡
Emergency	90 (15.2)	47 (17.6)	0.43‡
ASA PS > 2	232 (39.2)	69 (25.8)	<0.001‡
N_2O	522 (88.2)	254 (95.1)	0.002‡
Inhalation anesthetics	581 (98.1)	265 (99.2)	0.365*
Rocuronium, mg	100.0 (50.0, 100.0)	100.0 (100.0, 100.0)	<0.001†
Surgery time, hour	1.7 (1.0, 2.3)	1.3 (0.8, 2.0)	<0.001†
Intraoperative fluid, L	1.4 (0.7, 2.1)	0.7 (0.3, 1.3)	<0.001†
Estimated blood loss, L	0.5 (0.1, 1.0)	0.2 (0.0, 0.5)	<0.001†
Arterial line monitoring	415 (70.1)	154 (57.7)	<0.001‡
Central venous line monitoring	15 (2.5)	1 (0.4)	0.029*
Foley catheter	456 (77.0)	155 (58.0)	<0.001‡
Patient controlled analgesia	433 (73.1)	239 (89.5)	<0.001‡
Intraoperative PRBC, unit	2.0 (0.0, 4.0)	1.0 (0.0, 3.0)	<0.001†
BUN > 23mg·dl^{-1}	48 (8.1)	7 (2.6)	0.002*
Creatinine > 1.2mg·dl^{-1}	38 (6.4)	4 (1.5)	0.001*
aPTT > 40 seconds	144 (24.3)	75 (28.1)	0.277‡
PT, INR > 1.2	99 (16.7)	18 (6.7)	<0.001‡
Platelet <50000·$\mu\ell^{-1}$	11 (1.9)	0 (0)	0.021*
Albumin < 3.5g·dl^{-1}	81 (13.7)	9 (3.4)	<0.001*
Abdomen surgery	5 (0.8)	0 (0)	0.332*
Musculoskeletal surgery	67 (11.3)	46 (17.2)	0.024‡
Neurosurgery	1 (0.2)	1 (0.4)	0.525*
OBGY surgery	1 (0.2)	0 (0)	>0.999*

Spine surgery	1 (0.2)	0 (0)	>0.999*
Thoracic surgery	6 (1.0)	0 (0)	0.185*
Vascular surgery	0 (0)	1 (0.4)	0.311*
Skin & soft tissue surgery	577 (97.5)	258 (96.6)	0.506*
Major operation surgery	4 (0.7)	5 (1.9)	0.146*
Period from burn to surgery, day	4.0 (2.0, 8.0)	6.0 (3.0, 10.0)	0.001†
Intraoperative NSIADs	10 (1.7)	1 (0.4)	0.113‡
NSAIDs in patient controlled analgesia	175 (29.6)	21 (7.9)	<0.001*
Duration of postop. AAP, day	2 (0, 5)	1 (0, 3)	<0.001†
Duration of postop. Nefopam, day	0 (0,0)	0 (0,0)	0.274†
Duration of postop. NSAIDs, day	0 (0, 3.8)	2 (2.7)	<0.001†
Start date, day	0.0 (0.0, 0.0)	0.0 (0.0, 0.0)	<0.001†
Stop date, day	10.0 (1.6, 14.8)	12.9 (7.0, 18.4)	<0.001†

Data are presented as numbers (%) or medians (interquartile range)

AAP, acetaminophen; aPTT, activated partial thromboplastin time; ASA PS, American Society of Anesthesiologists physical status; body mass index; BUN, blood urea nitrogen; INR, international normalized ratio; n, number of surgical cases; NSAID, non-steroidal anti-inflammatory drug; OBGY, obstetric and gynecological; PRBC, packed red blood cell; PT, prothrombin time

† Start date, start of observation based on the date of first surgery (observation start date – date of first surgery);

†† Stop date, end of observation based on the date of first surgery (observation stop date – date of first surgery)

‡, chi-square test; *, Fisher's exact test; †, Mann-Whitney test

Supplementary table 6. Demographic characteristics and perioperative data in surgical cases of patients with severe burn who underwent surgery at 14 and more days from burn injury

	Nonuse of sugammadex (n = 1018)	Sugammadex (n =382)	P value
Old Age (≥70 years)	50 (4.9)	31 (8.1)	0.031‡
Male	881 (86.5)	342 (89.5)	0.159‡
Obesity (BMI≥30)	27 (2.6)	21 (5.5)	0.015‡
Congestive heart failure	9 (0.9)	1 (0.3)	0.302*
Cardiac arrhythmias	8 (0.8)	8 (2.1)	0.05*
Valvular disease	3 (0.3)	1 (0.3)	>0.999*
Pulmonary circulation Disorders	1 (0.1)	1 (0.3)	0.471*
Peripheral vascular disorders	7 (0.7)	0 (0)	0.2*
Hypertension, uncomplicated	86 (8.4)	36 (9.4)	0.638‡
Hypertension, complicated	32 (3.1)	5 (1.3)	0.062*
Paralysis	4 (0.4)	2 (0.5)	0.667*
Other neurological disorders	5 (0.5)	1 (0.3)	>0.999*
Chronic pulmonary disease	10 (1.0)	0 (0)	0.071*
Diabetes, uncomplicated	16 (1.6)	12 (3.1)	0.098‡
Diabetes, complicated	229 (22.5)	37 (9.7)	<0.001‡
Hypothyroidism	1 (0.1)	0 (0)	>0.999*
Renal failure	5 (0.5)	3 (0.8)	0.457*
Liver disease	27 (2.6)	17 (4.5)	0.122‡
Peptic ulcer disease excluding bleeding	6 (0.6)	0 (0)	0.198*
Coagulopathy	0 (0)	0 (0)	>0.999‡
Fluid and electrolyte disorders	0 (0)	0 (0)	>0.999‡
Deficiency anemia	2 (0.2)	0 (0)	>0.999*
Alcohol abuse	1 (0.1)	0 (0)	>0.999*
Drug abuse	4 (0.4)	2 (0.5)	0.667*
Psychoses	6 (0.6)	4 (1.1)	0.475*
Depression	140 (13.8)	66 (17.3)	0.116‡
Alcohol	542 (53.2)	218 (57.1)	0.222‡
Smoking	406 (39.9)	144 (37.7)	0.494‡
Emergency	6 (0.6)	1 (0.3)	0.681*
ASA PS > 2	39 (3.8)	33 (8.6)	<0.001‡
N_2O	992 (97.5)	372 (97.4)	>0.999‡
Inhalation anesthetics	955 (93.8)	378 (99.0)	<0.001*
Rocuronium, mg	50.0 (50.0, 100.0)	100.0 (100.0, 100.0)	<0.001†
Surgery time, hour	1.2 (0.7, 1.8)	1.2 (0.8, 1.8)	0.3†
Intraoperative fluid, L	0.7 (0.3, 1.2)	0.4 (0.2, 0.8)	<0.001†
Estimated blood loss, L	0.2 (0.0, 0.5)	0.1 (0.0, 0.2)	<0.001†
Arterial line monitoring	293 (28.8)	126 (33.0)	0.143‡
Central venous line monitoring	2 (0.2)	0 (0)	>0.999*
Foley catheter	328 (32.2)	134 (35.1)	0.342‡
Patient controlled analgesia	847 (83.2)	322 (84.3)	0.683‡
Intraoperative PRBC, unit	0.0 (0.0, 0.0)	0.0 (0.0, 1.0)	0.889†
BUN > $23mg{\cdot}dl^{-1}$	15 (1.5)	13 (3.4)	0.037‡
Creatinine > $1.2mg{\cdot}dl^{-1}$	17 (1.7)	13 (3.4)	0.074‡
aPTT > 40 seconds	214 (21.0)	126 (33.0)	<0.001‡
PT, INR > 1.2	72 (7.1)	12 (3.1)	0.008‡
Platelet <$50000{\cdot}\mu\ell^{-1}$	1 (0.1)	0 (0)	>0.999*
Albumin < $3.5g{\cdot}dl^{-1}$	2 (0.2)	0 (0)	>0.999*
Abdomen surgery	2 (0.2)	1 (0.3)	>0.999*
Musculoskeletal surgery	206 (20.2)	81 (21.2)	0.745‡
Neurosurgery	1 (0.1)	2 (0.5)	0.183*
OBGY surgery	0 (0)	0 (0)	>0.999‡

Spine surgery	0 (0)	0 (0)	>0.999‡
Thoracic surgery	0 (0)	0 (0)	>0.999‡
Vascular surgery	2 (0.2)	0 (0)	>0.999*
Skin & soft tissue surgery	968 (95.1)	368 (96.3)	0.395‡
Major operation surgery	29 (2.9)	15 (3.9)	0.391‡
Period from burn to surgery, day	31.0 (19.2, 98.0)	30.0 (19.0, 65.0)	0.09†
Intraoperative NSIADs	32 (3.1)	5 (1.3)	0.057‡
NSAIDs in patient controlled analgesia	449 (44.1)	37 (9.7)	<0.001‡
Duration of postop. AAP, day	0 (0, 2)	0 (0, 2)	0.375†
Duration of postop. Nefopam, day	0 (0, 1)	0, (0, 0)	0.163†
Duration of postop. NSAIDs, day	1 (0, 4)	3 (0, 6)	<0.001†
Start date, day	14.9 (0.0, 47.8)	20.1 (10.0, 46.7)	0.004†
Stop date, day	37.5 (20.6, 87.5)	39.5 (24.8, 71.3)	0.428†

Data are presented as numbers (%) or medians (interquartile range)

AAP, acetaminophen; aPTT, activated partial thromboplastin time; ASA PS, American Society of Anesthesiologists physical status; body mass index; BUN, blood urea nitrogen; INR, international normalized ratio; n, number of surgical cases; NSAID, non-steroidal anti-inflammatory drug; OBGY, obstetric and gynecological; PRBC, packed red blood cell; PT, prothrombin time

† Start date, start of observation based on the date of first surgery (observation start date – date of first surgery);

†† Stop date, end of observation based on the date of first surgery (observation stop date – date of first surgery)

‡, chi-square test; *, Fisher's exact test; ‖, Mann-Whitney test

Supplementary table 7. Unadjusted and adjusted hazard ratio on developing postoperative pulmonary complications in patients with severe burn who underwent within 14 days from burn injury.

	Unadjusted HR (95% CI)	P value	Adjusted HR (95% CI)	P value
Old Age (≥70 years)	1.59 (1.19–2.12)	0.002	1.72 (1.11–2.66)	0.016
Male	0.83 (0.62–1.12)	0.232	0.73 (0.5–1.07)	0.11
Obesity (BMI≥30)	1.58 (0.94–2.66)	0.086	1.12 (0.62–2.02)	0.7
Congestive heart failure	3.74 (1.54–9.08)	0.003	2.53 (0.77–8.26)	0.125
Cardiac arrhythmias	2.04 (0.96–4.33)	0.062	2.66 (1–7.12)	0.051
Valvular disease	1.3 (0.32–5.24)	0.709	1.46 (0.18–12.13)	0.726
Pulmonary circulation Disorders	1.15 (0.16–8.18)	0.891	0 (0–Inf)	0.999
Peripheral vascular disorders	0 (0–Inf)	0.99	47.99 (0–Inf)	>0.999
Hypertension, uncomplicated	1.09 (0.72–1.65)	0.696	0.67 (0.35–1.27)	0.219
Hypertension, complicated	1.6 (0.79–3.23)	0.191	1.62 (0.67–3.94)	0.284
Paralysis	1.07 (0.27–4.28)	0.928	0.15 (0.01–1.6)	0.117
Other neurological disorders	2.98 (0.95–9.29)	0.061	3.6 (0.97–13.36)	0.055
Chronic pulmonary disease	0 (0–Inf)	0.992	0 (0–Inf)	0.997
Diabetes, uncomplicated	0.71 (0.29–1.72)	0.448	0.92 (0.25–3.35)	0.898
Diabetes, complicated	0.9 (0.64–1.26)	0.527	1.13 (0.73–1.75)	0.588
Hypothyroidism	2.02 (0.28–14.37)	0.484	3.12 (0.38–25.63)	0.29
Renal failure	2.76 (1.03–7.4)	0.044	1.33 (0.35–4.98)	0.677
Liver disease	0.9 (0.4–2.02)	0.797	1.56 (0.64–3.78)	0.327
Peptic ulcer disease excluding bleeding	0 (0–Inf)	0.993	0 (0–Inf)	0.997
Coagulopathy	4.41 (1.64–11.89)	0.003	0.64 (0.21–2)	0.446
Fluid and electrolyte disorders	2.42 (0.34–17.27)	0.377	13170000 (0–Inf)	0.999
Deficiency anemia	0 (0–Inf)	0.991	0 (0–Inf)	0.998
Alcohol abuse	0 (0–Inf)	0.99	0 (0–Inf)	0.999
Drug abuse	1.39 (0.44–4.33)	0.573	1.28 (0.32–5.1)	0.726
Psychoses	1.18 (0.29–4.74)	0.816	0.95 (0.21–4.3)	0.952
Depression	0.73 (0.49–1.08)	0.115	0.67 (0.42–1.06)	0.083
Alcohol	0.98 (0.77–1.24)	0.865	1.17 (0.86–1.59)	0.314
Smoking	1 (0.79–1.28)	0.997	0.93 (0.69–1.25)	0.623
Emergency	1.85 (1.4–2.46)	<0.001	1.21 (0.84–1.74)	0.312
ASA PS > 2	4.49 (3.51–5.74)	<0.001	1.39 (1.01–1.91)	0.044
N2O	0.34 (0.25–0.47)	<0.001	0.78 (0.53–1.16)	0.22
Inhalation anesthetics	0.61 (0.25–1.49)	0.281	0.64 (0.23–1.81)	0.402
Rocuronium	1.01 (1–1.01)	0.004	1 (0.99–1)	0.289
Surgery time, hour	1.11 (1.02–1.21)	0.018	1.01 (0.89–1.14)	0.899
Fluid, Liter	1.21 (1.16–1.25)	<0.001	1.05 (0.92–1.19)	0.489
Estimated blood loss, Liter	2.21 (1.93–2.52)	<0.001	0.9 (0.63–1.28)	0.542
Arterial line monitoring	12.75 (7.45–21.84)	<0.001	4.08 (1.97–8.46)	<0.001
Central venous line monitoring	1.68 (0.79–3.56)	0.176	1.18 (0.51–2.72)	0.701
Foley catheter	10.19 (5.95–17.45)	<0.001	1.35 (0.69–2.64)	0.377
Patient controlled analgesia	0.36 (0.28–0.46)	<0.001	0.91 (0.65–1.27)	0.589
Intraoperative PRBC, unit	1.33 (1.28–1.38)	<0.001	1.06 (0.95–1.18)	0.303

Sugammadex	0.32 (0.22–0.45)	<0.001	0.61 (0.4–0.92)	0.019
BUN > 23mg·dl-1	3.25 (2.32–4.55)	<0.001	1.27 (0.79–2.03)	0.321
Creatinine > 1.2mg·dl-1	3.63 (2.5–5.25)	<0.001	1.25 (0.75–2.1)	0.396
aPTT > 40 seconds	1.83 (1.43–2.35)	<0.001	1 (0.73–1.36)	0.984
PT, INR > 1.2	3.24 (2.48–4.24)	<0.001	1.16 (0.83–1.63)	0.375
Platelet <50000·$\mu\ell^{-1}$	5.19 (2.66–10.11)	<0.001	1.08 (0.49–2.39)	0.842
Albumin < 3.5g·dl-1	6.36 (4.85–8.34)	<0.001	1.58 (1.08–2.31)	0.018
Abdomen surgery	2.4 (0.77–7.49)	0.132	2.8 (0.82–9.55)	0.1
Musculoskeletal surgery	0.61 (0.4–0.93)	0.022	0.99 (0.55–1.79)	0.979
Neurosurgery	2.45 (0.34–17.45)	0.372	603.9 (48.42–7533)	<0.001
OBGY surgery	0 (0–Inf)	0.993	0 (0–0)	>0.999
Spine surgery	2.9 (0.41–20.66)	0.289	9243000000 (0–Inf)	0.992
Thoracic surgery	9.51 (4.2–21.54)	<0.001	1.83 (0.69–4.88)	0.227
Vascular surgery	0 (0–Inf)	0.99	0 (0–Inf)	0.999
Skin & soft tissue surgery	0.97 (0.46–2.05)	0.935	0.9 (0.31–2.61)	0.843
Major operation surgery	0.33 (0.05–2.32)	0.262	0 (0–Inf)	0.993
head and neck (2nd grade / 3rd grade)	1.27 (1.14–1.4)	<0.001	1 (0.88–1.14)	0.964
trunk (2nd grade / 3rd grade)	1.33 (1.21–1.45)	<0.001	1.13 (1–1.28)	0.051
shoulder and upper limb, except wrist and hand (2nd grade / 3rd grade)	1.04 (0.95–1.14)	0.365	1.08 (0.94–1.23)	0.284
wrist and hand (2nd grade / 3rd grade)	0.93 (0.85–1.01)	0.094	0.95 (0.84–1.07)	0.397
hip and lower limb, except ankle and foot (2nd grade / 3rd grade)	1.12 (1.02–1.22)	0.014	0.98 (0.88–1.1)	0.794
ankle and foot (2nd grade / 3rd grade)	1.01 (0.91–1.12)	0.875	1.04 (0.92–1.18)	0.519
eye and adnexa	1.1 (0.35–3.44)	0.867	5.33 (1.21–23.52)	0.027
respiratory tract	2.63 (1.73–4.01)	<0.001	1.72 (1.01–2.92)	0.045
other internal organs	2.04 (0.29–14.52)	0.478	1.6 (0.13–20)	0.717
Period from burn to surgery, day	0.83 (0.79–0.86)	<0.001	0.93 (0.88–0.99)	0.012
Chemical burn	0.13 (0.05–0.34)	<0.001	0.75 (0.25–2.26)	0.607
Electrical burn	0.28 (0.2–0.39)	<0.001	0.97 (0.62–1.53)	0.892
Burn size grade	1.46 (1.39–1.52)	<0.001	1.12 (1.03–1.23)	0.012
Intraoperative NSIADs	0.52 (0.13–2.08)	0.352	0.63 (0.15–2.69)	0.532
NSAIDs in patient-controlled analgesia	0.86 (0.65–1.15)	0.306	1.12 (0.77–1.62)	0.566
Duration of postoperative AAP, day	1.06 (1.03–1.09)	<0.001	1.03 (0.99–1.07)	0.125
Duration of postoperative Nefopam, day	0.83 (0.73–0.94)	0.003	1.09 (0.98–1.21)	0.101
Duration of postoperative NSAIDs, day	0.8 (0.75–0.84)	<0.001	0.9 (0.85–0.95)	<0.001

AAP, acetaminophen; aPTT, activated partial thromboplastin time; ASA PS, American Society of

Anesthesiologists physical status; BMI, body mass index; BUN, blood urea nitrogen; CI, confidence interval; HR, hazard ratio; Inf, infinity; INR, international normalized ratio; n, number of surgical cases; NMB, neuromuscular blocker; NSAID, non-steroidal anti-inflammatory drug; OBGY, obstetric and gynecological; PRBC, packed red blood cell; Preop., preoperative; PT, prothrombin time

Burn size grade: 0 (<10%) ~ 9 (90–99%)

Supplementary table 8. Unadjusted and adjusted hazard ratio on developing postoperative pulmonary complications in patients with severe burn who underwent at 14 and more days from burn injury.

	Unadjusted HR (95% CI)	P value	Adjusted HR (95% CI)	P value
Old Age (≥70 years)	3.64 (1.66–7.95)	0.001	1.24 (0.28–5.58)	0.779
Male	0.69 (0.31–1.5)	0.344	1.04 (0.34–3.18)	0.945
Obesity (BMI≥30)	0.94 (0.13–6.83)	0.949	0.96 (0.12–7.61)	0.969
Congestive heart failure	0 (0–Inf)	0.997	0.45 (0–57.58)	0.75
Cardiac arrhythmias	2.95 (0.4–21.54)	0.286	1.26 (0.06–25.95)	0.88
Valvular disease	0 (0–Inf)	0.997	0.62 (0–881.8)	0.896
Pulmonary circulation Disorders	10.95 (1.49–80.39)	0.019	6.11 (0.22–170.2)	0.287
Peripheral vascular disorders	4.27 (0.57–31.79)	0.157	2.28 (0.07–74.79)	0.643
Hypertension, uncomplicated	2.1 (0.93–4.78)	0.075	1.13 (0.3–4.34)	0.857
Hypertension, complicated	1.71 (0.41–7.17)	0.462	1.26 (0.15–10.6)	0.829
Paralysis	8.91 (1.22–65.28)	0.031	3.97 (0.12–131.8)	0.44
Other neurological disorders	0 (0–Inf)	0.997	0.55 (0–2157)	0.889
Chronic pulmonary disease	0 (0–Inf)	0.997	0.6 (0–71.61)	0.833
Diabetes, uncomplicated	1.49 (0.2–10.88)	0.696	1.02 (0.08–12.7)	0.988
Diabetes, complicated	1.61 (0.8–3.24)	0.184	0.99 (0.41–2.42)	0.983
Hypothyroidism	0 (0–Inf)	0.998	0.63 (0–9002)	0.925
Renal failure	4.14 (0.57–30.18)	0.161	1.43 (0.02–93.03)	0.866
Liver disease	1.16 (0.16–8.47)	0.885	1.15 (0.11–11.74)	0.906
Peptic ulcer disease excluding bleeding	0 (0–Inf)	0.997	0.51 (0–66.97)	0.785
Deficiency anemia	23.94 (3.2–179.3)	0.002	25.92 (0.82–814.5)	>0.999
Alcohol abuse	0 (0–Inf)	0.997	1.11 (0–4164000)	0.989
Drug abuse	0 (0–Inf)	0.996	1 (0–416.7)	>0.999
Psychoses	11.99 (2.85–50.38)	<0.001	2.29 (0.09–59.14)	0.618
Depression	1.44 (0.63–3.27)	0.39	1.18 (0.42–3.29)	0.757
Alcohol	0.45 (0.23–0.86)	0.016	0.9 (0.42–1.93)	0.792
Smoking	0.86 (0.45–1.65)	0.649	1.06 (0.49–2.31)	0.884
Emergency	0 (0–Inf)	0.997	0.82 (0–147.1)	0.939
ASA PS > 2	5.54 (2.59–11.83)	<0.001	1.25 (0.29–5.37)	0.761
N2O	0.48 (0.12–2.01)	0.317	0.83 (0.1–6.69)	0.858
Inhalation anesthetics	27203334 (0–Inf)	0.996	1.13 (0.22–5.75)	0.882
Rocuronium	1.01 (1–1.01)	0.006	1 (0.99–1.01)	0.919
Surgery time, hour	1.02 (0.78–1.32)	0.907	0.98 (0.73–1.32)	0.919
Fluid, Liter	1.8 (1.46–2.23)	<0.001	1.18 (0.56–2.5)	0.658
Estimated blood loss, Liter	2.43 (1.66–3.56)	<0.001	0.72 (0.18–2.91)	0.64
Arterial line monitoring	5.42 (2.62–11.2)	<0.001	1.03 (0.4–2.65)	0.95
Central venous line monitoring	0 (0–Inf)	0.997	0.69 (0–2542)	0.929
Foley catheter	4.68 (2.27–9.62)	<0.001	0.94 (0.36–2.44)	0.892
Patient controlled analgesia	0.41 (0.19–0.87)	0.02	0.96 (0.3–3.06)	0.944
Intraoperative PRBC, unit	1.43 (1.29–1.58)	<0.001	1.1 (0.77–1.58)	0.584
Sugammadex	0.82 (0.38–1.79)	0.619	0.91 (0.36–2.33)	0.842
BUN > 23mg·dl^{-1}	5.48 (1.66–18.06)	0.005	1.95 (0.21–18.37)	0.559

Creatinine > 1.2mg·dl^{-1}	3.42 (0.82–14.26)	0.091	0.86 (0.05–15.59)	0.92
aPTT > 40 seconds	2.46 (1.3–4.66)	0.006	1 (0.43–2.33)	0.996
PT, INR > 1.2	8 (4.06–15.73)	<0.001	1.7 (0.53–5.51)	0.376
Platelet <50000·$\mu\ell^{-1}$	0 (0–Inf)	0.997	1.09 (0–11560000)	0.992
Albumin < 3.5g·dl^{-1}	161.6 (26.61–981.4)	<0.001	0.05 (0–251000000000000)	0.871
Abdomen surgery	0 (0–Inf)	0.997	1.19 (0–2529)	0.965
Musculoskeletal surgery	1.36 (0.61–3.01)	0.45	1.12 (0.39–3.2)	0.827
Vascular surgery	0 (0–Inf)	0.998	0.71 (0–1953000)	0.964
Skin & soft tissue surgery	9366663 (0–Inf)	0.996	1.16 (0.1–13.93)	0.905
Major operation surgery	1.22 (0.29–5.11)	0.784	0.71 (0.09–5.83)	0.746
head and neck (2nd grade / 3rd grade)	1.25 (0.94–1.65)	0.12	1.09 (0.76–1.55)	0.642
trunk (2nd grade / 3rd grade)	1 (0.76–1.31)	0.99	0.97 (0.68–1.37)	0.856
shoulder and upper limb, except wrist and hand (2nd grade / 3rd grade)	0.7 (0.51–0.98)	0.035	0.95 (0.68–1.34)	0.766
wrist and hand (2nd grade / 3rd grade)	0.81 (0.63–1.03)	0.084	0.97 (0.73–1.29)	0.832
hip and lower limb, except ankle and foot (2nd grade / 3rd grade)	1.33 (1.05–1.69)	0.019	1.02 (0.74–1.4)	0.925
ankle and foot (2nd grade / 3rd grade)	1.11 (0.84–1.45)	0.472	1 (0.73–1.39)	0.979
eye and adnexa	0 (0–Inf)	0.997	0.92 (0.04–20.34)	0.96
respiratory tract	0 (0–Inf)	0.995	0.86 (0.1–7.73)	0.893
Period from burn to surgery, day	1 (1–1)	0.48	1 (1–1)	0.992
Chemical burn	0.28 (0.04–2.1)	0.218	0.85 (0.17–4.22)	0.846
Electrical burn	0.35 (0.17–0.71)	0.004	0.9 (0.3–2.7)	0.845
Burn size grade	1.3 (1.1–1.52)	0.002	0.97 (0.7–1.34)	0.84
Intraoperative NSIADs	2.2 (0.53–9.16)	0.277	0.96 (0.12–7.91)	0.973
NSAIDs in patient-controlled analgesia	0.56 (0.28–1.14)	0.109	1 (0.42–2.37)	0.994
Duration of postoperative AAP, day	1.12 (1.06–1.19)	<0.001	1.02 (0.9–1.15)	0.792
Duration of postoperative Nefopam, day	1.08 (0.97–1.19)	0.175	1.02 (0.88–1.17)	0.816
Duration of postoperative NSAIDs, day	0.94 (0.87–1.03)	0.183	0.99 (0.91–1.07)	0.779

AAP, acetaminophen; aPTT, activated partial thromboplastin time; ASA PS, American Society of Anesthesiologists physical status; BMI, body mass index; BUN, blood urea nitrogen; CI, confidence interval; HR, hazard ratio; Inf, infinity; INR, international normalized ratio; n, number of surgical cases; NMB, neuromuscular blocker; NSAID, non-steroidal anti-inflammatory drug; OBGY, obstetric and gynecological; PRBC, packed red blood cell; Preop., preoperative; PT, prothrombin time

Burn size grade: 0 (<10%) ~ 9 (90–99%)

Supplementary table 9. The number of patients according to type and number of postoperative complications

	Patients with severe burn who underwent surgery				
**Type of PPC**	Within 14 days (n = 783)	14 and more days (n = 753)	All (n = 1213)	**Number of PPC**	PPC patients (n = 313)
Atelectasis	147 (18.8)	19 (2.5)	166 (13.7)	1	255 (81.5)
Pulmonary oedema)	28 (3.6)	6 (0.8)	34 (2.8)	2	51 (16.3)
Pleural effusion	137 (17.5)	12 (1.6)	149 (12.3)	3	3 (1.0)
Pneumothorax	1 (0.1)	1 (0.1)	2 (0.2)	4	2 (0.6)
Pneumonia	19 (2.4)	3 (0.4)	22 (1.8)	5	1 (0.3)
Pulmonary thromboembolism	1 (0.1)	2 (0.3)	3 (0.2)	6	1 (0.3)
Respiratory failure	3 (0.4)	2 (0.3)	5 (0.4)		
Acute respiratory distress syndrome	4 (0.5)	0 (0.0)	4 (0.3)		

PPC, postoperative pulmonary complication

Data is expressed as number (%)

Supplementary table 10. Number of patients by number of surgeries

Number of surgeries	Patients with severe burn who underwent surgery (n = 1213)			Patients with severe burn who underwent surgery or patients with major surgery (n = 1251)
	All	Surgeries within 14 days (n = 783)	Surgeries at 14 and more days (n = 753)	
1	748 (61.0)	717 (91.6)	456 (60.6)	748 (59.8)
2	241 (18.6)	57 (7.3)	149 (19.8)	241 (19.3)
3	122 (9.5)	8 (1.0)	68 (9.0)	122 (9.8)
4	65 (5.0)	1 (0.1)	26 (3.5)	65 (5.2)
5	25 (1.8)	0 (0)	24 (3.2)	25 (2.0)
6	16 (1.3)	0 (0)	9 (1.2)	16 (1.3)
7	11 (0.9)	0 (0)	13 (1.7)	11 (0.9)
8	12 (1.0)	0 (0)	4 (0.5)	12 (1.0)
9	6 (0.5)	0 (0)	1 (0.1)	6 (0.5)
10	3 (0.2)	0 (0)	1 (0.1)	3 (0.2)
11	0 (0)	0 (0)	2 (0.3)	0 (0)
12	1 (0.1)	0 (0)	0 (0)	1 (0.1)
13	1 (0.1)	0 (0)	0 (0)	1 (0.1)

Supplementary table 11. Diagnostic code and name of burn

KCD Code	Diagnosis
T20	Burn and corrosion of head and neck
T200	Burn of unspecified degree of head and neck
T201	Burn of first degree of head and neck
T202	Burn of second degree of head and neck
T203	Burn of third degree of head and neck
T21	Burn and corrosion of trunk
T210	Burn of unspecified degree of trunk
T211	Burn of first degree of trunk
T212	Burn of second degree of trunk
T213	Burn of third degree of trunk
T22	Burn and corrosion of shoulder and upper limb, except wrist and hand
T220	Burn of unspecified degree of shoulder and upper limb, except wrist and hand
T221	Burn of first degree of shoulder and upper limb, except wrist and hand
T222	Burn of second degree of shoulder and upper limb, except wrist and hand
T223	Burn of third degree of shoulder and upper limb, except wrist and hand
T23	Burn and corrosion of wrist and hand
T230	Burn of unspecified degree of wrist and hand
T231	Burn of first degree of wrist and hand
T232	Burn of second degree of wrist and hand
T233	Burn of third degree of wrist and hand
T24	Burn and corrosion of hip and lower limb, except ankle and foot
T240	Burn of unspecified degree of hip and lower limb, except ankle and foot
T241	Burn of first degree of hip and lower limb, except ankle and foot
T242	Burn of second degree of hip and lower limb, except ankle and foot
T243	Burn of third degree of hip and lower limb, except ankle and foot
T25	Burn and corrosion of ankle and foot
T250	Burn of unspecified degree of ankle and foot
T251	Burn of first degree of ankle and foot
T252	Burn of second degree of ankle and foot
T253	Burn of third degree of ankle and foot
T26	Burn and corrosion confined to eye and adnexa
T260	Burn of eyelid and periocular area
T261	Burn of cornea and conjunctival sac
T262	Burn with resulting rupture and destruction of eyeball
T263	Burn of other parts of eye and adnexa
T264	Burn of eye and adnexa, part unspecified
T27	Burn and corrosion of respiratory tract
T270	Burn of larynx and trachea

T271	Burn involving larynx and trachea with lung
T272	Burn of other parts of respiratory tract
T273	Burn of respiratory tract, part unspecified
T28	Burn and corrosion of other internal organs
T280	Burn of mouth and pharynx
T281	Burn of esophagus
T282	Burn of other parts of alimentary tract
T283	Burn of internal genitourinary organs
T284	Burn of other and unspecified internal organs
T29	Burns and corrosions of multiple body regions
T290	Burns of multiple regions, unspecified degree
T291	Burns of multiple regions, no more than first-degree burns mentioned
T292	Burns of multiple regions, no more than second-degree burns mentioned
T293	Burns of multiple regions, at least one burn of third degree mentioned
T30	Burn and corrosion, body region unspecified
T300	Burn of unspecified body region, unspecified degree
T301	Burn of first degree, body region unspecified
T302	Burn of second degree, body region unspecified
T303	Burn of third degree, body region unspecified
T31	Burns classified according to extent of body surface involved
T310	Burns involving less than 10% of body surface
T311	Burns involving 10-19% of body surface
T312	Burns involving 20-29% of body surface
T313	Burns involving 30-39% of body surface
T314	Burns involving 40-49% of body surface
T315	Burns involving 50-59% of body surface
T316	Burns involving 60-69% of body surface
T317	Burns involving 70-79% of body surface
T318	Burns involving 80-89% of body surface
T319	Burns involving 90% or more of body surface

KCD, Korean Standard Classification of Diseases (https://www.kcdcode.kr/browse/main/, accessed on September 19, 2022)

Supplementary table 12. Methods used to determine pulmonary complications.

Complication	Method
Atelectasis	Chest X-ray
Pulmonary edema	Chest X-ray
Pleural effusion	Chest X-ray
Pneumothorax	Chest X-ray
Pulmonary embolism	Chest computed tomography
Respiratory failure	Ventilator dependence for ≥2 postoperative days
Pneumonia	Pneumonia or infiltration with fever (>38 °C), white blood cell count < 4000/ μL or > 12000/μL or positive blood culture
Acute respiratory distress	Diagnosis based on respiratory medical consultation

Supplementary table 13. Definitions of burn characteristics

**Characteristics of burn**	Definition
Head and neck (2nd grade / 3rd grade)	KCD code: T202/T203
Trunk (2nd grade / 3rd grade)	KCD code: T212/T213
Shoulder and upper limb, except wrist and hand (2nd grade / 3rd grade)	KCD code: T222/T223
Wrist and hand (2nd grade / 3rd grade)	KCD code: T232/T233
Hip and lower limb, except ankle and foot (2nd grade / 3rd grade)	KCD code: T242/T243
Ankle and foot (2nd grade / 3rd grade)	KCD code: T252/T253
Eye and adnexa	KCD code: T26X
Respiratory tract	KCD code: T27X
Other internal organs	KCD code: T28X
Burn size	KCD code: T310, T311, T312, T313, T314, T315, T316, T317, T318, T319<br>OR check burn size in burn assessment record.
Chemical burn	Check chemical burn in burn assessment record.
Electrical burn	Check electrical burn in burn assessment record.

KCD, Korean Standard Classification of Diseases (https://www.kcdcode.kr/browse/main/, accessed on September 19, 2022)

Supplementary table 14. Definitions of demographic characteristics and perioperative covariates

**Patient's characteristics**	
Old age	≥70 years
Male	Male
Obesity	Body mass index ≥30
Congestive heart failure	KCD code: I09.9, I11.0, I13.0, I13.2, I25.5, I42.0, I42.5- I42.9, I43.x, I50.x, P29.0
Cardiac arrhythmias	KCD code: I44.1-I44.3, I45.6, I45.9, I47.X-I49.X, R00.0, R00.1, R00.8, T82.1, Z45.0, Z95.0
Valvular disease	KCD code: A52.0, I05.X-I08.X, I09.1, I09.8, I34.x-I39.x, Q23.0, Q23.3, Z95.2-Z95.4
Pulmonary circulation disorders	KCD code: I26.x, I27.x, I28.0, I28.8, I28.9
Peripheral vascular disorders	KCD code: I70.x, I71.x, I73.1, I73.8, I73.9, I77.1, I79.0, I79.2, K55.1, K55.8, K55.9, Z95.8, Z95.9
Hypertension, uncomplicated	KCD code: 110.x
Hypertension, complicated,	KCD code: I11.x-I13.x, I15.x
Paralysis	KCD code: G04.1, G 11.4, G80.1, G80.2, G81.x, G82.x, G83.0-G83.4, G83.9
Other neurological disorders	KCD code: G10.x-G13.x, G20.x-G22.x, G25.4, G25.5, G31.2, G31.8, G31.9, G32.x, G35.x-G37.x, G40.x, G41.X, G93.1, G93.4, R47.0, R56.x
Chronic pulmonary disease	KCD code: 127.8, 127.9, J40.X-J47.X, J60.X-J67.X, J68.4, J70.1, J70.3
Diabetes, uncomplicated	KCD code: E10.0, E10.1, E10.9, E11.0, E11.1, E11.9, E12.0, E12.1, E12.9, E13.0, E13.1, E13.9, EI4.0, E14.1, E14.9
Diabetes, complicated	KCD code: E10.2-E10.8, E11.2-E11.8, E12.2- E12.8. E13.2-E13.8, E14.2-E14.8
Hypothyroidism	KCD code: E00.X-E03.X, E89.0
Renal failure	KCD code: 112.0, 113.1, N18.x, N19.x, N25.0. Z49.0, Z49.2, Z94.0, Z99.2
Liver disease	KCD code: B18.x, I85.x, I86.4, I98.2, K70.x, K71.1, K71.3-K71.5, K71.7, K72.x- K74.x, K76.0, K76.2 K76.9, Z94.4
Peptic ulcer disease excluding bleeding	KCD code: K.25.7, K.25.9, K26.7, K.26.9, K27.7, K.27.9, K28.7, K28.9
Coagulopathy	KCD code: D65-D68.x, D69.1, D69.3- D69.6
Fluid and electrolyte disorders	KCD code: E22.2, E86.x, E87.x
Deficiency anaemia	KCD code: D50.8, D50.9, D51.x-D53.x
Alcohol abuse	KCD code: F10, E52, G62.1, I42.6, K29.2, K70.0, K70.3, K70.9, T51.x, Z50.2, Z71.4, Z72.1
Drug abuse	KCD code: F11.x-F16.x, F18.x, F19.x, Z71.5, Z72.2
Psychoses	KCD code: F20.x, F22.x-F25.x, F28.x, F29.x, F30.2, F31.2, F31.5
Depression	KCD code: F20.4, F31.3-F31.5, F32.x, F33.x, F34.1, F41.2, F43.2
Alcohol	On the electronic medical record, the patient's drinking status item.
Smoking	On the electronic medical record, the patient's smoking status item.
Emergency	Recorded as emergency surgery in pre-anaesthesia records.
ASA PS > 2	Recorded as American Society of Anaesthesiologists Physical Status Classification >2 in pre-anaesthesia records.
N_2O	Use of N_2O on prescription data for health insurance claims.
Inhalation anaesthetics	Use of inhalation anaesthetics on prescription data for health insurance claims.
Rocuronium	Administered dose of rocuronium during surgery

Arterial line monitoring	Use of arterial line monitoring on prescription data for health insurance claims
Central venous line monitoring	Use of central venous line monitoring on prescription data for health insurance claims
Foley catheter	Use of foley catheter on prescription data for health insurance claims
Patient controlled analgesia	Use of patient controlled analgesia on prescription data for health insurance claims
BUN > 23mg·dl^{-1}	Most recent preoperative test result for blood urea nitrogen >23mg·dl^{-1}
Creatinine > 1.2mg·dl^{-1}	Most recent preoperative test result for creatine>1.2mg·dl^{-1}
aPTT > 40 seconds	Most recent preoperative test result for Activated partial thromboplastin time >40 seconds
PT, INR > 1.2	Most recent preoperative test result for prothrombin time (international normalized ratio) >1.2
Platelet <50000·$\mu\ell^{-1}$	Most recent preoperative test result for platelet <50,000·$\mu\ell^{-}$
Albumin < 3.5g·dl^{-1}	Most recent preoperative test result for albumin < 3.5g dl^{-1}
Abdomen surgery	Procedure classification code: Q025x, Q0292, Q126x, Q2533-Q2534, Q2537, Q259x, Q2630-Q2638, Q2671-Q2673, Q2679, Q2687-Q2688, Q2921-Q2926, Q2928, Q2982-Q2984, Q722x-Q723x, Q7342, Q7360-Q7371, Q7410, Q7561-Q7562, Q7564, Q7567, Q757x, QA67x, QA92x, P208x-P209x, Q244x-Q252x, Q2536, Q254x-Q257, Q260x-Q262x, Q2639, Q264x-Q266x, Q2676, Q2680, Q269x-Q291x, Q2927, Q293x-Q297x, Q2981, Q299x-Q306x, Q721x, Q724x-Q733x, Q7341, Q7351-Q7352, Q7372, Q738x-Q740x, Q742x-Q755x, Q7563, Q7565-Q7566, Q758x-Q779x, QA536, QA63x-QA64x, QA75x, QX706, QX891
Musculoskeletal surgery	Procedure classification code: N0408, N0571, N0587, N0704, N0708, N0715, N1583-N1584, N1711, N1715, N1721, N1725, N2070, N2710-N2711, N2716, N3710-N3712, N3716-N3717, N3720-N3722, N3726-N3727, N4710-N4712, N4716-N4717, N4720-N4722, N4726-N4727, N002x, N025x-N031x, N035x-N039x, N0402-N0407, N041x-N043x, N050x-N056x, N0572-N0584, N0588, N059x-N062x, N0631-N0635, N064x-N069x, N0700-N0703, N0705-N0706, N0709-N0710, N0714, N0717-N0719, N072x-N100x, N1581-N1582, N1585, N160x-N161x, N1714, N1717, N1724, N1727, N2071-N2079, N2712-N2715, N2717-N2719, N3713-N3715, N3718-N3719, N3723-N3725, N3728-N3279, N4713-N4715, N4718-N4719, N4723-N4725, N4728-N4729, NA28x, NY05x
Neurosurgery	Procedure classification code: S4621-S4622, S4634-S2639, S464x-S466x, S4671, S4681, S4684, S4694-S4696, S4704, S4706, S4708, S4713, S4721, S4733-S4737, S4760, S4780, S4799, S4801-S4803, S6692, S6694, S6696, N032x-N034x, S047x, S459x-S461x, S4625, S4670, S4682-S4683, S4685, S4705, S4707, S4709-S4712, S4722-S4732, S474x-S475x, S477x, S4792-S4798, S4805, S481x-S485x, S6691, S6693, S6695, SY62x-SY63x
OBGY surgery	Procedure classification code: R014x, R040x-R041x, R313x-R314x, R401x-R500x, RA31x, RA36x-RA38x, RA43x, RY54x, RZ56x
Spine surgery	Procedure classification code: N0455, N0466, N0471-N0480, N1460, N1469, N2464-N2470, N044x, N0451-N0454, N0468-

	N0469, N0630, N1466, N149x, N2461-N2463, N2471-N2472, N249x
Thoracic surgery	Procedure classification code: O130x, O1313-O1319, O132x-O162x O160x-O162x, Q233x-Q243x, QA42x
Vascular surgery	Procedure classification code: O016x-O017x, O0215-O2018, O0223-O0227, O026x-O028x, O1635, O1643-O1646, O165x, O201x-O208x, O265x, OA63x, OA65x, OB63x-OB64x, OX181, OZ201
Skin & soft tissue surgery	Procedure classification code: SA161-SA165, SC161-SC165, N001x, N004x-N024x, N113x, N151x, NA05x, NA24x, NX201, S016x-S017x, SB16x-SB17x, SB27x
Major operation surgery	Procedure classification code: Q025x, Q0292, Q126x, Q2533-Q2534, Q2537, Q259x, Q2630-Q2638, Q2671-Q2673, Q2679, Q2687-Q2688, Q2921-Q2926, Q2928, Q2982-Q2984, Q722x-Q723x, Q7342, Q7360-Q7371, Q7410, Q7561-Q7562, Q7564, Q7567, Q757x, QA67x, QA92x, N0408, N0571, N0587, N0704, N0708, N0715, N1583-N1584, N1711, N1715, N1721, N1725, N2070, N2710-N2711, N2716, N3710-N3712, N3716-N3717, N3720-N3722, N3726-N3727, N4710-N4712, N4716-N4717, N4720-N4722, N4726-N4727, S4621-S4622, S4634-S2639, S464x-S466x, S4671, S4681, S4684, S4694-S4696, S4704, S4706, S4708, S4713, S4721, S4733-S4737, S4760, S4780, S4799, S4801-S4803, S6692, S6694, S6696, N0455, N0466, N0471-N0480, N1460, N1469, N2464-N2470, SA161-SA165, SC161-SC165
Start date	The start date of observation relative to the date of first surgery.
Stop date	End date of observation relative to first surgery date.
Surgery time	Time from start to end of surgery
Fluid, L	Administered total fluid amount regardless kinds of fluid during surgery.
Estimated blood loss, L	The amount of intraoperative bleeding estimated by the anaesthesiologist.
Intraoperative Packed red blood cell, unit	Total amount of packed red blood cells administered during surgery
Period from burn to surgery, day	Period from burn injury date to surgery date
Intraoperative NSIADs	NSAIDs (Dexketoprofen, Diclofenac, Ibuprofen, Ketoprofen, Piroxicam potassium) administration during surgery
NSAIDs in patient controlled analgesia	Whether PCA includes NSAIDs (Dexketoprofen, Diclofenac, Ibuprofen, Ketoprofen, Piroxicam potassium)
Duration of postop. AAP, day	Number of days of acetaminophen administration after surgery
Duration of postop. Nefopam, day	Number of days of nefopam administration after surgery
Duration of postop. NSAIDs, day	Number of days of NSAIDs (Dexketoprofen, Diclofenac, Ibuprofen, Ketoprofen, Piroxicam potassium)administration after surgery

Procedure classification code (https://www.koicd.kr/ins/act.do, accessed on September 19, 2022)

AAP, acetaminophen; aPTT, activated partial thromboplastin time; ASA PS, American Society of Anesthesiologists physical status; BUN, blood urea nitrogen; INR, international normalized ratio; n, number of surgical cases; NMB, neuromuscular blocker; NSAID, non-steroidal anti-inflammatory drug; OBGY, obstetric and gynecological; PRBC, packed red blood cell; Preop., preoperative; PT, prothrombin time
